# Genomic selection using random regressions on known and latent environmental covariates

**DOI:** 10.1007/s00122-022-04186-w

**Published:** 2022-09-06

**Authors:** Daniel J. Tolhurst, R. Chris Gaynor, Brian Gardunia, John M. Hickey, Gregor Gorjanc

**Affiliations:** 1 The Roslin Institute and Royal (Dick) School of Veterinary Studies, University of Edinburgh, Easter Bush, United Kingdom; 2 Cotton Product Design, Bayer CropScience, St Louis, USA; 3 Corn Product Design, Bayer CropScience, Barcelona, Spain

## Abstract

**Key message:**

The integration of known and latent environmental covariates within a single-stage genomic selection approach provides breeders with an informative and practical framework to utilise genotype by environment interaction for prediction into current and future environments.

**Abstract:**

This paper develops a single-stage genomic selection approach which integrates known and latent environmental covariates within a special factor analytic framework. The factor analytic linear mixed model of Smith et al. (2001) is an effective method for analysing multi-environment trial (MET) datasets, but has limited practicality since the underlying factors are latent so the modelled genotype by environment interaction (GEI) is observable, rather than predictable. The advantage of using random regressions on known environmental covariates, such as soil moisture and daily temperature, is that the modelled GEI becomes predictable. The integrated factor analytic linear mixed model (IFA-LMM) developed in this paper includes a model for predictable and observable GEI in terms of a joint set of known and latent environmental covariates. The IFA-LMM is demonstrated on a late-stage cotton breeding MET dataset from Bayer CropScience. The results show that the known covariates predominately capture crossover GEI and explain 34.4% of the overall genetic variance. The most notable covariates are maximum downward solar radiation (10.1%), average cloud cover (4.5%) and maximum temperature (4.0%). The latent covariates predominately capture non-crossover GEI and explain 40.5% of the overall genetic variance. The results also show that the average prediction accuracy of the IFA-LMM is $0.02-0.10$ higher than conventional random regression models for current environments and $0.06-0.24$ higher for future environments. The IFA-LMM is therefore an effective method for analysing MET datasets which also utilises crossover and non-crossover GEI for genomic prediction into current and future environments. This is becoming increasingly important with the emergence of rapidly changing environments and climate change.

**Supplementary Information:**

The online version contains supplementary material available at 10.1007/s00122-022-04186-w.

## Introduction

This paper develops a single-stage genomic selection (GS) approach which integrates known and latent environmental covariates within a special factor analytic framework. The factor analytic linear mixed model of Smith et al. (2001) is an effective method for analysing multi-environment trial (MET) datasets, which includes a parsimonious model for genotype by environment interaction (GEI). The advantage of using random regressions on known environmental covariates, such as soil moisture and maximum temperature, is that the modelled GEI becomes predictable. The GS approach developed in this paper exploits the desirable features of both classes of model.

Genomic selection is a form of marker-assisted selection that can improve the genetic gain in animal and plant breeding programmes (Meuwissen et al. 2001). In plant breeding, however, GS is often restricted by the presence of GEI, that is the change in genotype response to a change in environment. There are two appealing features of using known environmental covariates for GS; (i) meaningful biological interpretation can be ascribed to GEI and (ii) predictions can be obtained for any tested or untested genotype into any current or future environment. These features represent two long-standing objectives of many plant breeding programmes.

Regressions on known environmental covariates were first used in plant breeding by Yates and Cochran (1938). Their approach was later popularised by Finlay and Wilkinson (1963), and includes a fixed coefficient regression on a set of environmental mean yields (covariates) with a separate intercept and slope for each genotype. Hardwick and Wood (1972) extended the fixed regression model to include a more complex set of environmental covariates, such as moisture and temperature (also see Wood 1976). These approaches have distinct limitations when used to analyse MET datasets, however (Smith et al. 2005). An alternative approach is to use a linear mixed model with a random coefficient regression. This approach was popularised by Laird and Ware (1982), and requires an appropriate variance model for the intercepts and slopes which ensures the regression is scale and translational invariant. Heslot et al. (2014) extended the random regression model for GS using a set of genotype covariates derived from marker data and a set of environmental covariates derived from weather data. They were unable to fit an appropriate variance model for the intercepts and slopes, however, so that the regression was not translational invariant. At a similar time, Jarquín et al. (2014) demonstrated an even simpler random regression model for a very large set of correlated environmental covariates. They found that the environmental covariates explained only 23% of the overall genetic variance. These examples highlight the current limitations of using known environmental covariates for GS. That is, they are often highly correlated and only explain a small proportion of GEI, and fitting an appropriate variance model is typically computationally prohibitive (Brancourt-Hulmel et al. 2000; Buntaran et al. 2021).

The factor analytic linear mixed model of Smith et al. (2001) includes a latent regression model for GEI in terms of a small number of common factors (also see Piepho 1997). This approach is a linear mixed model analogue to AMMI (Gauch 1992) and GGE (Yan et al. 2000), or more specifically factor analysis (Mardia et al. 1979), where the factors involve some combination of latent environmental covariates. It also bears similarities to the ordinary regression models with one important difference; the environmental covariates are estimated from the data as well as the genotype slopes. Several authors have discussed the addition of intercepts to the factor analytic model in an attempt to obtain a simple average (*simple* main effect) for each genotype, but note there are issues which limit their interpretability (Smith 1999).

The factor analytic linear mixed model has been widely adopted for the analysis of MET datasets (Ukrainetz et al. 2018). The two main variants involve pedigree or marker data (Oakey et al. 2007, 2016). Recently, Tolhurst et al. (2019) demonstrated a factor analytic linear mixed model for GS within a major Australian plant breeding programme. They demonstrated genomic selection tools to obtain a measure of overall performance (*generalised* main effect) and stability for each genotype (Smith and Cullis 2018). There is one limitation of this approach, however. The common factors are latent so the modelled GEI is observable, rather than predictable. This limitation has lead to *ad hoc* post processing of the latent factors with known covariates (Oliveira et al. 2020).

Until now, the analysis of MET datasets has involved only one set of known *or* latent environmental covariates. The aim of this paper is to extend the GS approach of Tolhurst et al. (2019) to integrate both known *and* latent environmental covariates. This new approach is hereafter referred to as the integrated factor analytic linear mixed model (IFA-LMM). There are three appealing features of the IFA-LMM:

1. The IFA-LMM includes a regression model for GEI in terms of a small number of known and latent common factors. This simultaneously reduces the dimension of the known and latent environmental covariates.
2. The regression model captures *predictable* GEI in terms of *known* covariates. This enables meaningful interpretation of GEI and genomic prediction into any current or future environment.
3. The regression model also captures *observable* GEI in terms of *latent* covariates, which are orthogonal to the known covariates. This enables the regression model to capture a large proportion of GEI overall, and thence enables the IFA-LMM to be an effective method for analysing MET datasets.

The IFA-LMM is demonstrated on a late-stage cotton breeding MET dataset from Bayer CropScience. The predictive ability of the IFA-LMM is compared to several popular random regression models.

## Materials and methods

The Bayer CropScience Cotton Breeding Programme evaluates the commercial merit of test genotypes by annually conducting multi-environment field trials. There are two late-stages of field evaluation considered in this paper, referred to as preliminary commercial P1 and P2. The 2017 P1 MET dataset comprises the *current* set of environments and will be used to train all random regression models. The 2018 P2 MET dataset will be used to assess the predictive ability into *future* environments.

### Data description

#### Experimental design and phenotypic data

**Fig. 1:**
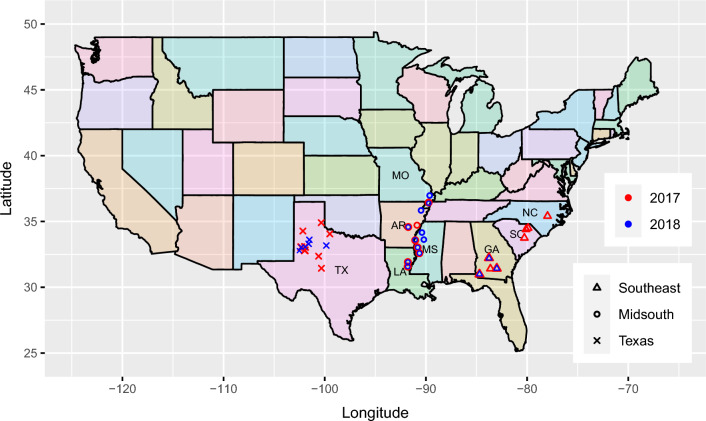
Map of the cotton growing environments in the 2017 P1 and 2018 P2 MET datasets. *Note*: States and years are distinguished by *colour* and growing regions are distinguished by *shape*

Table 1 presents a summary of the 2017 P1 MET dataset for seed cotton yield. There were 72 field trials conducted in 24 environments across eight states in Southeast, Midsouth and Texas, USA (Fig. 1). A total of 208 genotypes were evaluated in all environments. Each environment consisted of three trials. Each trial was designed as a randomised complete block design with 144 plots comprising two replicate blocks of 68 test genotypes plus four checks. Yield data were recorded on most plots with 6.54% missing. The number of non-missing plots per test genotype ranged from 39 to 47, with mean of 45. The number of non-missing genotypes in common between environments ranged from 173 to 208, with mean of 204. The mean yield and generalised narrow-sense heritability (Oakey et al. 2006) varied substantially between environments and growing regions.

**Table 1:** Summary of the 2017 P1 MET dataset for seed cotton yield

State	Env	Trials	Genotypes$^*$				Plots		Yield	
			Total	1rep	2rep	>2rep	Total	NAs	Mean	$h^2$
$\scriptstyle \triangle$ North carolina	17NC1	3	208	15	189	4	432	16	1.43	0.48
	17SC1	3	206	0	202	4	432	5	1.63	0.59
	17SC2	3	183	52	127	4	432	107	1.94	0.46
$\scriptstyle \triangle$ South carolina	17SC3	3	208	5	199	4	432	5	2.32	0.50
	17GA1	3	208	2	202	4	432	3	1.72	0.59
	17GA2	3	208	2	202	4	432	2	1.92	0.64
	17GA3	3	208	2	202	4	432	2	1.74	0.50
$\scriptstyle \triangle$ Georgia	17GA4	3	208	2	202	4	432	2	1.62	0.49
° Missouri	17MO1	3	207	69	134	4	432	76	1.95	0.61
	17AR1	3	207	18	185	4	432	20	0.99	0.24
° Arkansas	17AR2	3	205	2	199	4	432	9	1.63	0.83
	17MS1	3	204	9	191	4	432	19	1.21	0.57
	17MS2	3	207	6	197	4	432	10	1.93	0.63
° Mississippi	17MS3	3	207	140	63	4	432	150	0.91	0.55
	17LA1	3	208	4	200	4	432	6	1.32	0.72
° Louisiana	17LA2	3	208	11	193	4	432	12	1.16	0.60
	17TX1	3	208	1	203	4	432	1	2.12	0.62
	17TX2	3	208	2	202	4	432	2	1.79	0.59
	17TX3	3	207	4	199	4	432	7	2.05	0.72
	17TX4	3	208	4	200	4	432	4	1.86	0.38
	17TX5	3	198	132	62	4	432	161	1.38	0.56
	17TX6	3	206	29	173	4	432	33	1.95	0.43
	17TX7	3	208	7	197	4	432	7	1.77	0.56
$\times$ Texas	17TX8	3	208	18	186	4	432	19	2.57	0.40
**Overall**	–	**72**	**208**	–	–	–	**10,368**	**678**	**1.70**	**0.55**

Presented for each environment is the number of trials, genotypes (with one, two or more replicates) and plots (total and missing), as well as the mean yield (t/ha) and generalised narrow-sense heritability ($h^2$)

*Note*: *Symbols* distinguish the $\scriptstyle \triangle$ Southeast, ° Midsouth and $\times$ Texas growing regions

^*^Total number after missing plots removed

Supplementary Table 9 presents a summary of the 2018 P2 MET dataset for seed cotton yield. There were 20 field trials conducted in 20 environments across six states of USA (Fig. 1). Eleven trials were conducted in the same locations as the 2017 P1 trials and nine were conducted in new locations. A total of 55 genotypes were evaluated in all trials, with all genotypes previously evaluated in 2017 P1. Each trial was designed as a completely randomised design with a single replicate of all 55 genotypes. Note that only three environments were harvested in the Southeast due to severe weather.

#### Environmental data

Table 2 and Supplementary Table 10 present a summary of the known environmental covariates in the 2017 P1 and 2018 P2 MET datasets. There were 18 covariates available for all 44 environments, including latitude and longitude as well as 11 covariates derived from daily weather data and 5 covariates derived from daily soil data. These tables show that the known covariates vary substantially within and between growing regions, as well as between years. Each covariate was then centred and scaled to unit length for all subsequent analyses. The practical implication of this will be discussed in  “Regressions on latent covariates”.

**Table 2:** Summary of the known environmental covariates in the 2017 P1 MET dataset

		$\triangle$ Southeast			$\circ$ Midsouth			$\times$ Texas		
Covariate	Description (units)	Min	Mean	Max	Min	Mean	Max	Min	Mean	Max
LAT	latitude ($^\circ$)	31.0	33.0	35.4	31.6	33.6	36.4	31.4	33.2	34.9
LONG	longitude ($^\circ$)	− 84.7	− 81.7	− 78.0	− 91.9	− 91.1	− 89.7	− 102.3	− 101.1	− 99.5
avgCCR	average cloud cover (%)	53.4	56.0	59.1	46.6	48.7	52.2	32.1	34.5	37.0
minHUM	min humidity (%)	43.7	47.7	53.7	52.0	53.4	55.9	30.1	34.0	40.4
maxDSR	max downward solar radiation (W/m$^2$)	0.74	0.76	0.77	0.75	0.76	0.77	0.82	0.85	0.87
maxNSR	max net solar radiation (W/m$^2$)	0.62	0.64	0.66	0.63	0.64	0.65	0.68	0.68	0.70
maxPRP	max precipitation (mm/hr)	2.4	2.9	3.4	1.7	2.6	3.6	1.1	1.4	1.8
totPRP	total precipitation (mm/day)	3.2	3.5	4.2	3.0	3.7	4.9	1.3	1.6	2.1
maxDPT	max dew point temperature ($^\circ$C)	20.5	21.1	22.1	18.9	20.7	22.0	13.5	15.7	17.6
maxTMP	max temperature ($^\circ$C)	28.5	30.3	31.5	27.6	28.9	29.6	28.7	30.3	32.1
minTMP	min temperature ($^\circ$C)	19.0	20.1	21.0	17.9	19.5	20.4	15.4	17.4	19.5
minWSP	min wind speed (km/hr)	4.9	5.2	5.7	4.7	4.9	5.0	7.4	8.1	9.4
avgWDR	average wind direction (azimuth degrees)	166.7	175.8	181.5	152.3	161.9	174.0	144.7	152.9	162.9
maxST1	max soil temperature 1 ($^\circ$C)	27.6	29.9	31.3	27.0	28.3	29.1	29.5	32.2	34.5
minST1	min soil temperature 1 ($^\circ$C)	19.8	21.8	23.2	19.3	20.6	21.5	19.0	20.6	22.8
avgSM3	soil moisture 3 (%)	7.0	23.8	42.3	28.0	30.1	32.7	11.4	19.0	25.6
avgSM4	soil moisture 4 (%)	10.0	29.5	44.6	29.8	32.9	35.3	8.3	15.8	21.8
minST4	min soil temperature 4 ($^\circ$C)	20.0	22.4	24.2	20.2	22.0	23.0	21.0	22.9	25.2

*Note:* Values presented are prior to centring and scaling

Presented for each covariate is the minimum, mean and maximum for the $\scriptstyle \triangle$ Southeast, $\circ$ Midsouth and $\times$ Texas growing regions

#### Marker data

Marker data were available for 204 (of the 208) genotypes in 2017 P1, which included all 55 genotypes in 2018 P2. The markers correspond to a high confidence set of 36,009 single-nucleotide polymorphisms. Genotypes were coded as either −1, 0 or 1 for the homozygous minor, heterozygous or homozygous major alleles at each marker. The frequency of heterozygous markers was low given the level of selfing accumulated up to the P1 stage. Monomorphic markers were then removed and missing markers were imputed using the *k*-nearest neighbour approach of Troyanskaya et al. (2001), with $k = 10$. Note that the four genotypes without marker data are of no practical interest (see Tolhurst et al. 2019, for further details).

The genomic relationship matrix was constructed using the *pedicure* package (Butler 2019) in *R* (R Core Team 2021). The default settings in *pedicure* were used as filters, with minor allele frequency > 0.002% and missing marker frequency < 0.998%. A total of 24,265 markers were retained using this criteria. The diagonal elements of the relationship matrix ranged from 0.004 to 2.022, with mean of 1.234. The off-diagonals ranged from −0.388 to 1.322, with mean of −0.006.

### Statistical models

#### Preliminaries

Assume the MET dataset comprises $v=204$ genotypes evaluated in $t=72$ field trials conducted across $p=24$ environments, where $t=\sum _{j=1}^p t_j$ and $t_j=3$ is the number of trials in environment *j*. Let the *n*-vector of phenotypic data be given by $\mathbf{y}=\big (\mathbf{y}_1^{\scriptscriptstyle \top },\mathbf{{y}}_2^{\scriptscriptstyle \top }, \ldots , \mathbf{{y}}_p^{\scriptscriptstyle \top }\big )^{\!\scriptscriptstyle \top }$, where $\mathbf{{y}}_j=\big (\mathbf{{y}}_{j;1}^{\scriptscriptstyle \top },\mathbf{{y}}_{j;2}^{\scriptscriptstyle \top }, \ldots , \mathbf{{y}}_{j;t_j}^{\scriptscriptstyle \top }\big )^{\!\scriptscriptstyle \top }$ is the $n_j$-vector for environment *j* and $\mathbf{{y}}_{j;k}$ is the $n_{jk}$-vector for trial *k* in environment *j*. The length of $\mathbf{{y}}$ is therefore given by:

$$\begin{aligned} n = \sum _{j=1}^{p} \sum _{k=1}^{t_j} n_{jk} = \sum _{j=1}^p n_{j}. \end{aligned}$$

Lastly, assume all $p=24$ environments have $q=18$ known covariates available, that is assume $p>q$. Let the $p\times q$ matrix of covariates be given by $\mathbf{{S}}=\big[\mathbf{{s}}_1 \ \mathbf{{s}}_2 \ \ldots \ \mathbf{{s}}_q\big]$, with columns given by the centred and scaled environment scores for each covariate, such that $\mathbf{{s}}_i^{\scriptscriptstyle \top }\mathbf{{s}}_i=1.$

#### Linear mixed model

The linear mixed model for $\mathbf{{y}}$ can be written as:

1 $$\begin{aligned} \mathbf{{y}} = \mathbf{{X}}\varvec{\tau } + \mathbf{{Z}}\mathbf{{u}} + \mathbf{{Z_p}}\mathbf{{u_p}} +\mathbf{{e}}, \end{aligned}$$

where $\varvec{\tau }$ is a vector of fixed effects with design matrix $\mathbf{{X}}$, $\mathbf{{u}}$ is a *vp*-vector of random *genotype by environment* (GE) effects with $n \times vp$ design matrix $\mathbf{{Z}}$, $\mathbf{{u_p}}$ is a vector of random non-genetic peripheral effects with design matrix $\mathbf{{Z_p}}$ and $\mathbf{{e}}$ is the *n*-vector of residuals.

The vector of fixed effects, $\varvec{\tau }$, includes the mean parameter for each environment. This vector is fitted as fixed following a classical quantitative genetics approach where the GE effects in different environments are regarded as different correlated traits (Falconer and Mackay 1996). This vector can be extended to a regression on known environmental covariates, with:

2 $$\begin{aligned} \varvec{\tau }=\mathbf{{1}}_p\mu + \mathbf{{S}}{\varvec{\tau }}_\mathbf{{s}}+\varvec{\omega }, \end{aligned}$$

where *μ* is the overall mean parameter (intercept), $\mathbf{{S}}$ is the $p\times q$ matrix of known covariates, $\varvec{\tau} _{\mathbf{{s}}}$ is a *q*-vector with elements given by the mean response of genotypes to each covariate and $\varvec{\omega }$ is a *p*-vector of residual environmental effects, with $\varvec{\omega} \sim \text{ N }\big(\mathbf{{0}}, \sigma^2_\omega \mathbf{{I}}_p\big)$.

The vector of random non-genetic effects, $\mathbf{{u_p}}$, accommodates the plot structures of trials and environments (Bailey 2008). This vector is fitted as random to enable recovery of information across incomplete blocks and trials (Patterson and Thompson 1971). Other effects in $\mathbf{{u_p}}$ may accommodate extraneous variations across field columns and rows (Gilmour et al. 1997).

It is assumed that:

3 $$\begin{aligned} \left[ \begin{array}{c} \mathbf{{u}} \\ \mathbf{{u_p}} \\ \mathbf{{e}} \end{array}\right] \sim \text{ N }\left( \left[ \begin{array}{c} \mathbf{{0}} \\ \mathbf{{0}} \\ \mathbf{{0}} \end{array}\right] , \left[ \begin{array}{ccc} \mathbf{{G}} &{} \mathbf{{0}} &{} \mathbf{{0}} \\ \mathbf{{0}} &{} \, \mathbf{{G_p}} &{} \mathbf{{0}} \\ \mathbf{{0}} &{} \mathbf{{0}} &{} \, \mathbf{{R}} \end{array}\right] \right) . \end{aligned}$$

Following Tolhurst et al. (2019), $\mathbf{{G_p}} = \oplus _{j=1}^p \mathbf{{G}}_{\mathbf{{p}}_j}$ is diagonal with a separate variance component model for the *j*^*th*^ environment and $\mathbf{{R}} = \oplus _{j=1}^p \mathbf{{R}}_{j}$ is block diagonal with a two-dimensional spatial model for the *j*^*th*^ environment. The form of $\mathbf{{G}}$ is presented below, but note that all variance matrices in Eq. 3 are fitted at the environment level, not trial level. This completely aligns the non-genetic and residual variance models with the genetic variance model.

### Variance model for the GE effects

The GE effects are modelled using $r=24,265$ markers, and therefore referred to as the *additive* GE effects. This model is an extension of the univariate GBLUP model (Stranden and Garrick 2009), with:

4 $$\begin{aligned} \mathbf{{u}} = \big (\mathbf{{I}}_p \otimes \mathbf{{M}}\big )\mathbf{{u_m}} \qquad \text {and} \qquad \mathbf{{G}}&= \mathbf{{G_e}} \otimes \mathbf{{M}}{\mathbf{{M}}}^{\scriptscriptstyle \top }/m \nonumber \\&= \mathbf{{G_e}} \otimes \mathbf{{G_g}}, \end{aligned}$$

where $\mathbf{{M}} = \big[\mathbf{{m}}_1 \ \mathbf{{m}}_2 \ \ldots \ \mathbf{{m}}_r\big]$ is a $v\times r$ design matrix with columns given by the centred genotype scores for each marker, $\mathbf{{u_m}}$ is a *rp*-vector of additive *marker by environment* effects, $\mathbf{{G_e}}$ is a $p\times p$ additive genetic variance matrix between environments and $\mathbf{{G_g}} = \mathbf{{M}}\mathbf{{M}}^{\scriptscriptstyle \top }/m$ is the $v\times v$ genomic relationship matrix between genotypes (VanRaden 2008).

The random regression models for $\mathbf{{u}}$ considered in this paper include:

1. Latent covariates; models with simple or generalised main effects.
2. Known covariates; models with or without translational invariance.
3. Known and latent covariates; models with generalised main effects and translational invariance.

All regression models are summarised in Table 3, with full details provided below.

### Regressions on latent covariates

The factor analytic model is effective for modelling the covariances between additive GE effects in terms of a small number of latent common factors (Kelly et al. 2007). The two variants considered in this paper include simple or generalised main effects.

#### Models with simple main effects

Smith et al. (2001) demonstrated an extension of the factor analytic model which includes an explicit intercept for each genotype. This extension will be referred to as the FAM*k* model, where *k* denotes the number of *latent* factors. The FAM*k* model is given by:

5 $$\begin{aligned} \mathbf{{u}}&= \big (\mathbf{{1}}^\star _p \otimes \mathbf{{I}}_v \big ) \varvec{\gamma} _{\mathbf{{1}}} + \big (\varvec{\lambda }_{1} \otimes \mathbf{{I}}_v \big ) \mathbf{{f}}_{1} + \ldots + \big (\varvec{\lambda }_{k} \otimes \mathbf{{I}}_v \big ) \mathbf{{f}}_{k} + \varvec{\delta } \nonumber \\&= \big ({\mathbf{{1}}}^\star _p \otimes \mathbf{{I}}_v \big ) \varvec{\gamma} _{\mathbf{{1}}} + \big (\varvec{\Lambda } \otimes \mathbf{{I}}_v \big ) \mathbf{{f}} + \varvec{\delta }, \end{aligned}$$

with $\mathbf{{1}}^\star _p=\mathbf{{1}}_p/\sqrt{p}$, where $\varvec{\gamma}_{\mathbf{{1}}} = \big({{\gamma}}_{1_1}, {{\gamma}}_{1_2},\ldots , {{\gamma}}_{1_v}{\big)}^{\!\scriptscriptstyle \top }$ is a *v*-vector of genotype intercepts, $\varvec{\Lambda } = \big [\varvec{\lambda }_{1} \, \varvec{\lambda }_{2} \, \ldots \, \varvec{\lambda }_{k}\big ]$ is a $p\times k$ matrix of latent environmental loadings (covariates), ${\mathbf{{f}}} = \big(\mathbf{{f}}_{1}^{{\scriptscriptstyle \hskip -0.5pt \top }}, {\mathbf{{f}}}_{2}^{{\scriptscriptstyle \hskip -0.5pt \top }},\ldots , {\mathbf{{f}}}_{k}^{{\scriptscriptstyle \hskip -0.5pt \top }}{\big)}^{\!\scriptscriptstyle \top }$ is a *vk*-vector of genotype scores (slopes) in which $\mathbf{{f}}_{l}$ is the *v*-vector for the $l^{th}$ latent factor and $\varvec{\delta } = \big({\varvec{\delta }}_{1}^{{\scriptscriptstyle \top }}, {\varvec{\delta }}_{2}^{{\scriptscriptstyle \top }},\ldots , {\varvec{\delta }}_{p}^{{\scriptscriptstyle \top }}{\big)}^{\!\scriptscriptstyle \top }$ is a *vp*-vector of regression residuals (deviations) in which $\varvec{\delta }_{j}$ is the *v*-vector specific to the $j^{th}$ environment. This specification highlights the analogy to an ordinary random regression, with the difference that the environmental covariates are estimated from the data as well as the genotype slopes (see Eq. 13).

Following Smith et al. (2021), the loadings are assumed to have orthonormal columns, with ${\varvec{\Lambda }}^{\!\scriptscriptstyle \top }\varvec{\Lambda }=\mathbf{{I}}_{k}$, and the scores are assumed to be independent across factors, with non-unit variance. It therefore follows that:

$$\begin{aligned} \left[ \begin{array}{c} \varvec{\gamma} _{\mathbf{{1}}} \\ \mathbf{{f}} \\ \varvec{\delta } \end{array}\right] \sim \text{ N }\left( \left[ \begin{array}{c} \mathbf{{0}} \\ \mathbf{{0}} \\ \mathbf{{0}} \end{array}\right] , \left[ \begin{array}{ccc} p\sigma _1^2 &{} \mathbf{{0}} &{} \mathbf{{0}}\\ \mathbf{{0}} &{} \ \mathbf{{D}} \ \text {} &{} \mathbf{{0}} \\ \mathbf{{0}} &{} \mathbf{{0}} &{} \varvec{\Psi } \end{array}\right] \otimes \mathbf{{G_g}} \right) , \end{aligned}$$

where $\sigma _1^2$ is the intercept variance, $\mathbf{{D}}=\oplus _{l=1}^k d_l$ is a diagonal matrix in which $d_{l}$ is the score variance for the $l^{th}$ latent factor ordered as $d_1>d_2>\ldots >d_k$ and $\varvec{\Psi } = \oplus _{j=1}^p\psi _j$ is a diagonal matrix in which $\psi _{j}$ is the specific variance for the $j^{th}$ environment. The variance matrix for $\mathbf{{u}}$ is then given by:

6 $$\begin{aligned} \mathbf{{G}} = \left( \big[\mathbf{{1}}^\star _p \ \varvec{\Lambda }\big]\left[ \begin{array}{cc} p\sigma _1^2 &{} \, \mathbf{{0}} \\ \mathbf{{0}} &{} \, \mathbf{{D}} \end{array} \right] \big[\mathbf{{1}}^*_p \ \varvec{\Lambda }{\big]}^{\scriptscriptstyle \top }+ \varvec{\Psi }\right) \otimes \mathbf{{G_g}}, \end{aligned}$$

where $\mathbf{{G_e}} \equiv \sigma _1^2\mathbf{{J}}_p + \varvec{\Lambda} \mathbf{D}{\varvec{\Lambda }}^{\!\scriptscriptstyle \top }+ \varvec{\Psi }$ and $\mathbf{{J}}_p=\mathbf{{1}}_p{\mathbf{{1}}}_p^{{\scriptscriptstyle \top }}$. This variance matrix highlights the analogy to a random regression without translational invariance, that is where the intercepts and slopes are independent (see Eq. 14).

Note that the intercepts in $\varvec{\gamma}_{\mathbf{{1}}}$ reflect the fitted value of each genotype at zero values of the environmental loadings. In order for the intercepts to reflect true main effects, however, the average values of the loadings must also be zero. The analogy to ordinary regression models is when the known covariates are column centred, so that the intercepts will reflect main effects taken at average (zero) values of the covariates.

Smith (1999) use a Gram-Schmidt process to column centre the environmental loadings (see “Appendix”). The variance matrix in Eq. 6 can therefore be written as:

7 $$\begin{aligned} \mathbf{{G}} = \left( \big[\mathbf{{1}}^\star _p \ \varvec{\Lambda }^{\hskip -1pt \star} \big]\left[ \begin{array}{cc} p{\sigma }_g^{2} &{} \, {\mathbf{{D}}}^\star _{12} \\ {\mathbf{{D}}}^{\star}_{21} &{} \, {\mathbf{{D}}}^\star _{22} \end{array} \right] \big[\mathbf{{1}}^\star _p \ \varvec{\Lambda }^{\hskip -1pt \star} {\big]}^{{\scriptscriptstyle \top }} + \varvec{\Psi }\right) \otimes \mathbf{{G_g}}, \end{aligned}$$

where $\mathbf{G_e} \equiv \sigma_g^2\mathbf{J}_p + \mathbf{1}_p^{\star}{\mathbf{D}}^{\star}_{12}\boldsymbol{\Lambda}^{\star {\!\scriptscriptstyle \hskip 0.75pt \top}} + \boldsymbol{\Lambda}^{\star}{\mathbf{D}}^{\star}_{21}\mathbf{1}_p^{\star {\!\scriptscriptstyle \hskip 0.75pt \top}} +\boldsymbol{\Lambda }^{\star}{\mathbf{D}}^{\star}_{22}\boldsymbol{\Lambda}^{\star {\!\scriptscriptstyle \hskip 0.75pt \top}} +\boldsymbol{\Psi}$, with ${\varvec{\Lambda }}^{\hskip -1pt \star {\scriptscriptstyle \top }}{\mathbf{{1}}}_p^\star =\mathbf{{0}}$. This variance matrix highlights the analogy to a random regression with translational invariance, that is where the main effects and slopes are dependent (see Eq. 19). This variance matrix also highlights the analogy to a special FA($k+1$) model, where the first factor loadings are constrained to be equal and the higher order loadings sum to zero.

The *simple* main effects are now equivalent to simple averages across environments, with:

8 $$\begin{aligned} {\varvec{\gamma}}_{\mathbf{{g}}} = \varvec{\gamma}_{\mathbf{{1}}} + \sqrt{p}{\sum _{l=1}^k\bar{\lambda }_l}\mathbf{{f}}_l \quad \text {and} \quad {\varvec{\gamma }}_{\mathbf{{g}}} \sim \text{ N }\left( \mathbf{{0}}, \, p{\sigma }_g^{2} \mathbf{{G_g}}\right) , \end{aligned}$$

where ${\sigma }_g^{2}=\sigma _1^{2} + {\sum _{l=1}^kd_l{\bar{\lambda }}^2_l}$ is the simple main effect variance and $\bar{\lambda }_l={\mathbf{{1}}}_p^{\scriptscriptstyle \top }\varvec{\lambda }_l/p$ is the mean loading for the $l^{th}$ latent factor. The distinguishing feature compared to the intercepts in Eq. 5 is that the simple main effects now reflect the fitted value of each genotype at average (zero) values of the loadings.

The percentage of additive genetic variance explained by the simple main effects is given by:

9 $$\begin{aligned} {v}_g = 100\, p{\sigma }_g^{2}/\text {tr}(\mathbf{{G}}_{\mathbf{{e}}}), \end{aligned}$$

where $\mathbf{{G}}_{\mathbf{{e}}}$ is defined in Eq. 7.

#### Models with generalised main effects

The conventional factor analytic (FA*k*) model is a simplification of the FAM*k* model in Eq. 5, with:

10 $$\begin{aligned} \mathbf{{u}} = \big (\varvec{\Lambda } \otimes \mathbf{{I}}_v \big ) \mathbf{{f}} + \varvec{\delta } \ \quad \text {and} \ \quad \mathbf{{G}} = \big (\varvec{\Lambda} \mathbf{D}{\varvec{\Lambda }}^{\!\scriptscriptstyle \top }+ \varvec{\Psi }\big ) \otimes \mathbf{{G_g}}, \end{aligned}$$

where $\mathbf{{G}}_{\mathbf{{e}}}= \varvec{\Lambda} \mathbf{D}{\varvec{\Lambda }}^{\!\scriptscriptstyle \top }+ \varvec{\Psi }$. The distinguishing feature of this model is that intercepts are not explicitly fitted for each genotype (see “Appendix”).

Smith and Cullis (2018) discuss the ability of factor analytic models to capture heterogeneity of scale variance, that is non-crossover GEI, within the first factor. They proposed a set of *generalised* main effects based on this factor, with:

11 $$\begin{aligned} \varvec{\gamma}_{\mathbf{{g}}}^\star = \bar{\lambda }_1\mathbf{{f}}_1 \qquad \text {and} \qquad \varvec{\gamma}_{\mathbf{{g}}}^\star \sim \text{ N }\left( \mathbf{{0}}, \, d_1{\bar{\lambda }}_1^2 \mathbf{{G_g}}\right) , \end{aligned}$$

where $\bar{\lambda }_1={\mathbf{{1}}}_p^{\scriptscriptstyle \top }\varvec{\lambda }_1/p$ and $\varvec{\lambda }_1$ is the *p*-vector of first factor loadings which are assumed to be positive. The generalised main effects can therefore be viewed as weighted averages across environments. This highlights an important difference to the simple main effects in the FAM*k* model, which are simple averages across environments.

The percentage of additive genetic variance explained by the generalised main effects is equivalent to the variance explained by the first factor, which is given by:

12 $$\begin{aligned} {v}_1 = 100\, d_1/\text {tr}\big ({\mathbf{{G}}}_{\mathbf{{e}}}\big ), \end{aligned}$$

where $\mathbf{{G_e}}$ is defined in Eq. 10. This measure will be compared to the variance explained by the simple main effects in “Results”.

### Regressions on known covariates

The ordinary random regression model is given by:

13 $$\begin{aligned} \mathbf{{u}}&= \big (\mathbf{{1}}^\star_p \otimes \mathbf{{I}}_v \big ) \varvec{\gamma}_\mathbf{{g}} + \big (\mathbf{{s}}_{1} \otimes \mathbf{{I}}_v \big ) \varvec{\gamma }_{\mathbf{{s}}_1} + \ldots + \big (\mathbf{{s}}_{q} \otimes \mathbf{{I}}_v \big ) \varvec{\gamma }_{\mathbf{{s}}_q} + \varvec{\delta } \nonumber \\&= \big (\mathbf{{1}}^\star_p \otimes \mathbf{{I}}_v \big ) \varvec{\gamma}_\mathbf{{g}} + \big (\mathbf{{S}} \otimes \mathbf{{I}}_v \big ) \varvec{\gamma} _{\mathbf{{s}}} + \varvec{\delta }, \end{aligned}$$

where $\varvec{\gamma}_\mathbf{{g}} = \big({{\gamma}}_{g_1}, {{\gamma}}_{g_2},\ldots , {{\gamma}}_{g_v}{\big)}^{\!\scriptscriptstyle \top }$ is the *v*-vector of simple main effects, $\mathbf{{S}} = \big[\mathbf{{s}}_1 \ \mathbf{{s}}_2 \ \ldots \ \mathbf{{s}}_q\big]$ is the $p \times q$ matrix of centred and scaled known environmental covariates, $\varvec{\gamma}_\mathbf{{s}} = \big(\varvec{\gamma }_{\mathbf{{s}}_1}^{{\scriptscriptstyle \top }}, \varvec{\gamma }_{\mathbf{{s}}_2}^{{\scriptscriptstyle \top }},\ldots , \varvec{\gamma }_{\mathbf{{s}}_q}^{{\scriptscriptstyle \top }}{\big)}^{\!\scriptscriptstyle \top }$ is a *vq*-vector of genotype slopes in which $\varvec{\gamma }_{\mathbf{{s}}_i}$ is the *v*-vector for the $i^{th}$ known covariate and $\varvec{\delta } = \big({\varvec{\delta }}_{1}^{{\scriptscriptstyle \top }}, {\varvec{\delta }}_{2}^{{\scriptscriptstyle \top }},\ldots , {\varvec{\delta }}_{p}^{{\scriptscriptstyle \top }}{\big)}^{\!\scriptscriptstyle \top }$ is the *vp*-vector of regression residuals. This specification highlights the analogy to the FAM*k* model in Eq. 5. Note, however, that the known covariates are already column centred so that the intercepts already reflect simple main effects.

#### Models without translational invariance

The random regression model in Heslot et al. (2014) assumes independent main effects and slopes, with:

$$\begin{aligned} \left[ \begin{array}{c} \varvec{\gamma}_\mathbf{{g}} \\ \varvec{\gamma} _{\mathbf{{s}}} \end{array}\right] \sim \text{ N }\left( \left[ \begin{array}{c} \mathbf{{0}} \\ \mathbf{{0}} \end{array}\right] , \left[ \begin{array}{cc} p\sigma _g^2 &{} \mathbf{{0}} \\ \mathbf{{0}} &{} \, \varvec{\Sigma }_{\mathbf{{s}}} \end{array}\right] \otimes \mathbf{{G_g}}\right) , \end{aligned}$$

where $\sigma _g^2$ is the simple main effect variance and $\varvec{\Sigma }_{\mathbf{{s}}} = \oplus _{i=1}^{q}\sigma ^2_{s_i}$ is a diagonal matrix in which $\sigma ^2_{s_i}$ is the slope variance for the $i^{th}$ known covariate. The distributional assumption for $\varvec{\gamma}_{\mathbf{{s}}}$ may restrict interpretation, however, when the mean response to specific covariates is expected to be nonzero. The regression form of $\varvec{\tau }$ in Eq. 2 overcomes this issue, with $\varvec{\gamma}_{\mathbf{{s}}} \sim \text{ N }\big( \varvec{\tau}_{\mathbf{{s}}}\otimes \mathbf{{1}}_v, \varvec{\Sigma }_{\mathbf{{s}}} \otimes \mathbf{{G_g}}\big)$. The variance matrix for $\mathbf{{u}}$ is then given by:

14 $$\begin{aligned} \mathbf{{G}} = \left( \big[\mathbf{{1}}^\star _p \ \mathbf{{S}}\big]\left[ \begin{array}{cc} p\sigma _g^2 &{} \, \mathbf{{0}} \\ \mathbf{{0}} &{} \, \varvec{\Sigma }_{\mathbf{{s}}} \end{array} \right] \big[\mathbf{{1}}^\star _p \ \mathbf{{S}}{\big]}^{\scriptscriptstyle \top }+ \varvec{\Psi }\right) \otimes \mathbf{{G_g}}, \end{aligned}$$

where $$\mathbf{{G_e}} \equiv {\sigma }_g^2\mathbf{{J}}_p + \mathbf{{S}}\varvec{\Sigma }_{\mathbf{{s}}}{\mathbf{{S}}}^{\scriptscriptstyle \top }+ \varvec{\Psi }$$.

The random regression model in Jarquín et al. (2014) uses an even simpler variance matrix for the slopes, with $\text {var}(\varvec{\gamma} _{\mathbf{{s}}}) = \sigma _s^2\mathbf{{I}}_{q} \otimes \mathbf{{G_g}}$, where $\sigma _s^2$ is the slope variance across all known covariates. The variance matrix for $\mathbf{{u}}$ is then given by:

15 $$\begin{aligned} \mathbf{{G}} = \left( \big[\mathbf{{1}}^\star _p \ \mathbf{{S}}\big]\left[ \begin{array}{cc} p\sigma _g^2 &{} \, \mathbf{{0}} \\ \mathbf{{0}} &{} \, \sigma _s^2\mathbf{{I}}_{q} \end{array} \right] \big[\mathbf{{1}}^\star _p \ \mathbf{{S}}{\big]}^{\scriptscriptstyle \top }+ \varvec{\Psi }\right) \otimes \mathbf{{G_g}}, \end{aligned}$$

where $\mathbf{{G_e}} \equiv \sigma _g^2\mathbf{{J}}_p + \sigma _s^2\mathbf{{S}}{\mathbf{{S}}}^{\scriptscriptstyle \top }+ \varvec{\Psi }$. Note that this random regression is neither scale nor translational invariant.

#### Models with translational invariance

Jennrich and Schluchter (1986) proposed an extension of the random regression model which includes a factor analytic model for the known environmental covariates. This extension will be referred to as the FAR*k* model, where *k* denotes the number of *known* factors. The FAR*k* model for the simple main effects and slopes in Eq. 13 is given by:

16 $$\begin{aligned} \varvec{\gamma}_\mathbf{{g}} = \big (\varvec{\Lambda}_{\mathbf{{g}}} \otimes \mathbf{{I}}_v \big ) \mathbf{{f}} + \varvec{\delta}_{\mathbf{g}} \ \quad \text {and} \ \quad \varvec{\gamma}_{\mathbf{{s}}} = \big (\varvec{\Lambda}_{\mathbf{{s}}} \otimes \mathbf{{I}}_v \big ) \mathbf{{f}} + \varvec{\delta}_{\mathbf{s}}, \end{aligned}$$

where $\mathbf{{f}} = \big({\mathbf{{f}}}_{1}^{{\scriptscriptstyle \hskip -0.5pt \top }}, {\mathbf{{f}}}_{2}^{{\scriptscriptstyle \hskip -0.5pt \top }},\ldots , {\mathbf{{f}}}_{k}^{{\scriptscriptstyle \hskip -0.5pt \top }}{\big)}^{\!\scriptscriptstyle \top }$ is the *vk*-vector of genotype scores which correspond to the *k* known factors. The FAR*k* model constructs a joint regression across the main effects and slopes, with loadings given by:

$$\begin{aligned} \varvec{\Lambda}_{\mathbf{{g}}} = \left[\lambda _{{g}_1} \, {\lambda }_{{g}_2} \, \ldots \, {\lambda }_{{g}_{k}}\right] \ \quad \text {and} \ \quad \varvec{\Lambda}_{\mathbf{{s}}} = \left[\varvec{\lambda }_{\mathbf{{s}}_1} \, \varvec{\lambda }_{\mathbf{{s}}_2} \, \ldots \, \varvec{\lambda }_{\mathbf{{s}}_{k}}\right], \end{aligned}$$

where ${\varvec{\Lambda }}^{\scriptscriptstyle \top }_{\mathbf{{g}}}$ is a *k*-vector and $\varvec{\Lambda}_{\mathbf{{s}}}$ is a $q\times k$ matrix. The deviations in Eq. 16 are given by:

$$\begin{aligned} \varvec{\delta}_\mathbf{{g}} = \left( \delta _{g_1}, \delta _{g_2}, \ldots , \delta _{g_v} \right) ^{{\!\scriptscriptstyle \top }} \ \quad \text {and} \ \quad \varvec{\delta}_{\mathbf{{s}}} = \big ({\varvec{\delta }}^{\scriptscriptstyle \top }_{\mathbf{{s}}_1}, {\varvec{\delta }}^{\scriptscriptstyle \top }_{\mathbf{{s}}_2}, \ldots , {\varvec{\delta }}^{\scriptscriptstyle \top }_{\mathbf{{s}}_q} {\big )}^{{\!\scriptscriptstyle \top }}, \end{aligned}$$

where $\varvec{\delta}_\mathbf{{g}}$ is a *v*-vector and $\varvec{\delta}_{\mathbf{{s}}}$ is a *vq*-vector.

The inclusion of the deviations in Eq. 16 may be unnecessary, however, particularly for higher order FAR*k* models in which the percentage of variance explained by these effects is small. This leads to a reduced rank factor analytic model for the simple main effects and slopes (Kirkpatrick and Meyer 2004), with:

17 $$\begin{aligned} \varvec{\gamma}_{\mathbf{{g}}} = \big (\varvec{\Lambda}_{\mathbf{{g}}} \otimes \mathbf{{I}}_v \big ) \mathbf{{f}} \ \qquad \text {and} \ \qquad \varvec{\gamma}_{\mathbf{{s}}} = \big (\varvec{\Lambda}_{\mathbf{{s}}} \otimes \mathbf{{I}}_v \big ) \mathbf{{f}}. \end{aligned}$$

The main effects and slopes are assumed to be dependent, with:

$$\begin{aligned} \left[ \begin{array}{c} \varvec{\gamma}_{\mathbf{{g}}} \\ \varvec{\gamma}_{\mathbf{{s}}} \end{array}\right] \sim \text{ N }\left( \left[ \begin{array}{c} \mathbf{{0}} \\ \mathbf{{0}} \end{array}\right] , \left[ \begin{array}{cc} \varvec{\Lambda}_{\mathbf{{g}}}{\mathbf{{D}}}{\varvec{\Lambda }}^{\scriptscriptstyle \top }_{\mathbf{{g}}} &{} \, \varvec{\Lambda}_{\mathbf{{g}}}{\mathbf{{D}}}{\varvec{\Lambda }}^{\scriptscriptstyle \top }_{\mathbf{{s}}} \\ \varvec{\Lambda}_{\mathbf{{s}}}{\mathbf{{D}}}{\varvec{\Lambda }}^{\scriptscriptstyle \top }_{\mathbf{{g}}} &{} \, \varvec{\Lambda}_{\mathbf{{s}}}{\mathbf{{D}}}{\varvec{\Lambda }}^{\scriptscriptstyle \top }_{\mathbf{{s}}} \end{array}\right] \otimes \mathbf{{G_g}}\right) , \end{aligned}$$

where $\mathbf{{D}}=\oplus _{l=1}^k d_l$ is the score variance matrix with diagonal elements ordered as $d_1>d_2>\ldots >d_k$.

The FAR*k* model is then obtained by substituting the vectors in Eq. 17 into Eq. 13, which gives:

18 $$\begin{aligned} \mathbf{{u}}&= \left(\big[\mathbf{{1}}^\star _p\varvec{\Lambda}_{\mathbf{{g}}} + \mathbf{{S}}\varvec{\Lambda}_{\mathbf{{s}}}\big] \otimes \mathbf{{I}}_v \right) \mathbf{{f}} + \varvec{\delta }. \end{aligned}$$

The variance matrix for $\mathbf{{u}}$ is then given by:

19 $$\begin{aligned} \mathbf{{G}} = \left( \mathbf{{A}}\left[ \begin{array}{cc} \varvec{\Lambda}_{\mathbf{{g}}}{\mathbf{{D}}}{\varvec{\Lambda }}^{\scriptscriptstyle \top }_{\mathbf{{g}}} &{} \, \varvec{\Lambda}_{\mathbf{{g}}}{\mathbf{{D}}}{\varvec{\Lambda }}^{\scriptscriptstyle \top }_{\mathbf{{s}}} \\ \varvec{\Lambda}_{\mathbf{{s}}}{\mathbf{{D}}}{\varvec{\Lambda }}^{\scriptscriptstyle \top }_{\mathbf{{g}}} &{} \, \varvec{\Lambda}_{\mathbf{s}}{\mathbf{{D}}}{\varvec{\Lambda }}^{\scriptscriptstyle \top }_{\mathbf{{s}}}\end{array}\right] {\mathbf{{A}}}^{\!\scriptscriptstyle \top }+ \varvec{\Psi }\right) \otimes \mathbf{{G_g}}, \end{aligned}$$

where $\mathbf{{G_e}} \equiv \mathbf{{A}}\varvec{\Lambda} _\mathbf{{a}}\mathbf{{D}}{\varvec{\Lambda }}_{\mathbf{{a}}}^{\!\scriptscriptstyle \top }{\mathbf{{A}}}^{\!\scriptscriptstyle \top }+ \varvec{\Psi }$, $\mathbf{{A}} = [\mathbf{{1}}^\star _p \ \mathbf{{S}}]$ and $$\varvec{\Lambda} _\mathbf{{a}}=\Big [{\begin{array}{c}{{\varvec{\Lambda }}_{\mathbf{{g}}}} \\ {{\varvec{\Lambda }}_{\mathbf{{s}}}} \end{array}}\Big ]$$, with ${\varvec{\Lambda }}_{\mathbf{{a}}}^{\scriptscriptstyle\ \top }{\mathbf{{A}}}^{\!\scriptscriptstyle \top }\mathbf{{A}}\varvec{\Lambda }_{\mathbf{{a}}}=\mathbf{{I}}_{k}$. This variance matrix is equivalent to the conventional FA*k* variance matrix in Eq. 10 when $\mathbf{{A}}$ is square and has full rank.

### Regressions on known and latent covariates

The integrated factor analytic (IFA*k*) model is an extension of the FAR*k* model to include generalised main effects based on latent environmental covariates, instead of simple main effects. The IFA*k* model can also be viewed as a special FA*k* model with loadings constrained to be linear combinations of two orthogonal sources of GEI, that is known and latent environmental covariates. The loadings matrix in Eq. 5 can therefore be written as:

20 $$\begin{aligned} \varvec{\Lambda} &= \mathbf{S}\varvec{\Lambda}_\mathbf{s} + \varvec{\Gamma\Lambda}_\mathbf{r} & \hskip -2pt \quad \text{or} \qquad 
\varvec{\Lambda} &= \big[\mathbf{S}\varvec{\Lambda}_\mathbf{s} \, \ \varvec{\Gamma\Lambda}_\mathbf{r}\big]
 \nonumber \\ &= \mathbf{{B}}\left[ \begin{array}{c}\varvec{\Lambda}_\mathbf{s} \\ \varvec{\Lambda}_\mathbf{r} \end{array}\right]
 & &=\mathbf{{B}}\left[ \begin{array}{cc}\varvec{\Lambda}_\mathbf{s} & \mathbf{{0}} \\ \mathbf{{0}} & \varvec{\Lambda}_\mathbf{r} \end{array}\right], \end{aligned}$$

where $\mathbf{{B}} = [\mathbf{{S}} \ \, \varvec{\Gamma }]$ is a $p \times p$ matrix of basis functions which is assumed to have full rank,  $\mathbf{{S}} = \big[\mathbf{{s}}_1 \ \mathbf{{s}}_2 \ \ldots \ \mathbf{{s}}_q\big]$ is the $p\times q$ matrix of known environmental covariates and $\varvec{\Gamma }=\big[\mathbf{{r}}_1 \ \mathbf{{r}}_2 \ \ldots \ \mathbf{{r}}_{p-q}\big]$ is a $p\times (p-q)$ orthogonal projection matrix, with ${\mathbf{{S}}}^{\scriptscriptstyle \top }\varvec{\Gamma }=\mathbf{{0}}$. The two loadings matrices in Eq. 20 correspond to the dependent and independent formulations of the IFA*k* model. The dependent formulation is translational invariant, and thence the focus of this paper. No further reference will be made to the independent formulation, but full details are provided in the Supplementary Material.

The dependent formulation constructs a joint regression across the known and latent environmental covariates. The $p \times k$ matrix of joint factor loadings is given by:

21 $$\begin{aligned} \left[ \begin{array}{c}\varvec{\Lambda}_{\mathbf{{s}}} \\ \varvec{\Lambda }_{\mathbf{{r}}} \end{array}\right] = \left[ \begin{array}{c} \varvec{\lambda }_{\mathbf{{s}}_1} \, \varvec{\lambda }_{\mathbf{{s}}_2} \, \ldots \, \varvec{\lambda }_{\mathbf{{s}}_{k}} \\ \varvec{\lambda }_{\mathbf{{r}}_1} \, \varvec{\lambda }_{\mathbf{{r}}_2} \, \ldots \, \varvec{\lambda }_{\mathbf{{r}}_{k}} \end{array}\right] , \end{aligned}$$

where $\varvec{\Lambda}_\mathbf{s}$ is a $q\times k$ matrix corresponding to the known covariates and $\varvec{\Lambda}_\mathbf{r}$ is a $(p-q)\times k$ matrix corresponding to the latent covariates. The common factors underlying $\varvec{\Lambda}_\mathbf{s}$ and $\varvec{\Lambda}_\mathbf{r}$ are therefore referred to as the *known* and *latent* factors, and collectively as the *joint* factors.

The projection matrix in Eq. 20 is chosen to ensure that $\mathbf{{B}}$ has full rank and that the known and latent factors are orthogonal. This is achieved by projecting $\varvec{\Lambda}_\mathbf{r}$ into the orthogonal complement to the space spanned by $\mathbf{{S}}$. A convenient choice for $\varvec{\Gamma }$ is the first ($p-q$) columns in:

22 $$\begin{aligned} \big [\mathbf{{I}}_{p} - \mathbf{{S}}({\mathbf{{S}}}^{\scriptscriptstyle \top }\mathbf{{S}})^{-1}{\mathbf{{S}}}^{\scriptscriptstyle \top }\big ], \end{aligned}$$

assuming that *p* > *q*. This choice ensures that the same number of variance parameters are estimated as the conventional FA*k* model in Eq. 10. When $p\gg q$, however, it may be desirable to take fewer than $(p-q)$ columns in Eq. 22, and thence estimate fewer variance parameters. This enables the IFA*k* model to be scalable to a very large number of environments.

The IFA*k* model is obtained by substituting the first loadings matrix in Eq. 20 into Eq. 10, which gives:

23 $$\begin{aligned} \mathbf{{u}}&= \left (\big[\mathbf{{S}}\varvec{\Lambda} _\mathbf{s} + \varvec{\Gamma \Lambda}_\mathbf{r} \big] \otimes \mathbf{{I}}_v \right ) \mathbf{{f}} + \varvec{\delta }, \end{aligned}$$

where $\mathbf{{f}} = \big({\mathbf{{f}}}_{1}^{{\scriptscriptstyle \hskip -0.5pt\top }}, {\mathbf{{f}}}_{2}^{{\scriptscriptstyle \hskip -0.5pt \top }},\ldots , {\mathbf{{f}}}_{k}^{{\scriptscriptstyle \hskip -0.5pt \top }}{\big)}^{\!\scriptscriptstyle \top }$ is the *vk*-vector of genotype scores which correspond to the *k* joint factors.

The main difference to the FAR*k* model in Eq. 18 is that there are now two vectors of slopes, with:

24 $$\begin{aligned} \varvec{\gamma}_\mathbf{s} = \big (\varvec{\Lambda}_\mathbf{s} \otimes \mathbf{{I}}_v \big ) \mathbf{{f}} \ \qquad \text {and} \ \qquad \varvec{\gamma}_\mathbf{r} = \big (\varvec{\Lambda}_\mathbf{r} \otimes \mathbf{{I}}_v \big ) \mathbf{{f}}, \end{aligned}$$

where $\varvec{\gamma}_\mathbf{s}$ is a *vq*-vector corresponding to the known covariates and $\varvec{\gamma}_\mathbf{r}$ is a $v(p-q)$-vector corresponding to the latent covariates. Another important difference is the addition of generalised main effects in $\varvec{\gamma}_\mathbf{r}$, with:

25 $$\begin{aligned} \varvec{\gamma }^\star_{\mathbf{{g}}} = \bar{\lambda }_{{{r}}_1}\mathbf{{f}}_1 \ \qquad \text {and} \ \qquad \varvec{\gamma }^\star_{\mathbf{{g}}} \sim \text{ N }\left( \mathbf{{0}}, \, d_1{\bar{\lambda }}_{r_1}^2 \mathbf{{G_g}}\right) , \end{aligned}$$

where $\bar{\lambda }_{r_1}={\mathbf{{1}}}_{p-q}^{\scriptscriptstyle \top }\varvec{\lambda }_{\mathbf{{r}}_1}/p$. The IFA*k* model can therefore be viewed as a special random regression with generalised main effects as well as translational invariance.

The slopes in Eq. 24 are assumed to be dependent, with:

$$\begin{aligned} \left[ \begin{array}{c} \varvec{\gamma}_\mathbf{s} \\ \varvec{\gamma}_\mathbf{r} \end{array}\right] \sim \text{ N }\left( \left[ \begin{array}{c} \mathbf{{0}} \\ \mathbf{{0}} \end{array}\right] , \left[ \begin{array}{cc} \varvec{\Lambda}_\mathbf{s}\mathbf{D}{\varvec{\Lambda }}^{\scriptscriptstyle \top }_{\mathbf{{s}}} &{} \, \varvec{\Lambda}_\mathbf{s}\mathbf{D}{\varvec{\Lambda }}^{\scriptscriptstyle \top }_{\mathbf{{r}}} \\ \varvec{\Lambda}_\mathbf{r}\mathbf{D}{\varvec{\Lambda }}^{\scriptscriptstyle \top }_{\mathbf{{s}}} &{} \, \varvec{\Lambda}_\mathbf{r}\mathbf{D}{\varvec{\Lambda }}^{\scriptscriptstyle \top }_{\mathbf{{r}}} \end{array}\right] \otimes \mathbf{{G_g}}\right) , \end{aligned}$$

where $\mathbf{{D}}=\oplus _{l=1}^k d_l$ is the score variance matrix with diagonal elements ordered as $d_1>d_2> \ldots > d_k$. The variance matrix for $\mathbf{{u}}$ is then given by:

26 $$\begin{aligned} \mathbf{{G}} = \left( \mathbf{{B}}\left[ \begin{array}{cc} \varvec{\Lambda}_\mathbf{s}\mathbf{D}{\varvec{\Lambda }}^{\scriptscriptstyle \top }_{\mathbf{{s}}} &{} \, \varvec{\Lambda}_\mathbf{s}\mathbf{D}{\varvec{\Lambda }}^{\scriptscriptstyle \top }_{\mathbf{{r}}} \\ \varvec{\Lambda}_\mathbf{r}\mathbf{D}{\varvec{\Lambda }}^{\scriptscriptstyle \top }_{\mathbf{{s}}} &{} \, \varvec{\Lambda}_\mathbf{r}\mathbf{D}{\varvec{\Lambda }}^{\scriptscriptstyle \top }_{\mathbf{{r}}} \end{array} \right] {\mathbf{{B}}}^{\scriptscriptstyle \top }+ \varvec{\Psi }\right) \otimes \mathbf{{G_g}}, \end{aligned}$$

where $\mathbf{{G_e}} \equiv \varvec{\Lambda}\mathbf{D}{\varvec{\Lambda }}^{\!\scriptscriptstyle \top }+ \varvec{\Psi }$ and $$\varvec{\Lambda } = \mathbf{{B}}\Big [{\begin{array}{c}{{\varvec{\Lambda }}_{\mathbf{{s}}}} \\ {{\varvec{\Lambda }}_{\mathbf{{r}}}} \end{array}}\Big ]$$, with ${\varvec{\Lambda }}^{\!\scriptscriptstyle \top }\varvec{\Lambda }=\mathbf{{I}}_k$. This variance matrix is equivalent to the conventional FA*k* model in Eq. 10, where the factors are constrained to be linear combinations of known and latent environmental covariates.

### Model estimation

All variance models for the additive GE effects were implemented within the linear mixed model in Eq. 1. The two factor analytic linear mixed models with simple and generalised main effects are referred to as the FAM-LMM and FA-LMM, respectively. The other two linear mixed models developed in this paper are derived below.

The factor analytic regression linear mixed model (FAR-LMM) is obtained by substituting Eq. 18 into Eq. 1, which gives:

27 $$\begin{aligned} \mathbf{{y}} = \mathbf{{X}}\varvec{{\tau}} + \mathbf{{Z}}_{\varvec{\Lambda} _\mathbf{{a}}}\mathbf{{f}} + \mathbf{{Z}}\varvec{\delta } + \mathbf{{Z_p}}\mathbf{{u_p}} + \mathbf{{e}}, \end{aligned}$$

where $\mathbf{{Z}}_{\varvec{\Lambda} _\mathbf{{a}}}= \mathbf{{Z}}(\mathbf{{A}}\varvec{\Lambda}_\mathbf{{a}}\otimes \mathbf{{I}}_v)$. In this model, the covariances between the simple main effects and slopes are based on a reduced rank factor analytic model.

The integrated factor analytic linear mixed model (IFA-LMM) is obtained by substituting Eq. 23 into Eq. 1, which gives:

28 $$\begin{aligned} \mathbf{{y}} = \mathbf{{X}}\varvec{{\tau }} + \mathbf{{Z}}_{\varvec{\Lambda} _\mathbf{{b}}}\mathbf{{f}} + \mathbf{{Z}}\varvec{\delta } + \mathbf{{Z_p}}\mathbf{{u_p}} + \mathbf{{e}}, \end{aligned}$$

where $\mathbf{{Z}}_{\varvec{\Lambda} _\mathbf{{b}}} = \mathbf{{Z}}(\mathbf{{B}}\varvec{\Lambda }_\mathbf{{b}}\otimes \mathbf{{I}}_v)$ and $$\varvec{\Lambda}_\mathbf{{b}} = \Big [{\begin{array}{c}{{\varvec{\Lambda }}_{\mathbf{{s}}}} \\ {{\varvec{\Lambda }}_{\mathbf{{r}}}} \end{array}}\Big ]$$. In this model, the covariances between the known and latent environmental covariates are based on a reduced rank factor analytic model. The IFA-LMM will now be used to demonstrate all remaining methods. Similar results can be obtained for the other three linear mixed models where required.

#### Rotation of loadings and scores

Constraints are required in the IFA-LMM during estimation to ensure unique solutions for $$\Big [{\begin{array}{c}{{\varvec{\Lambda }}_{\mathbf{{s}}}} \\ {{\varvec{\Lambda }}_{\mathbf{{r}}}} \end{array}}\Big ]$$ and ${\mathbf{{D}}}$. Following Smith et al. (2021), the upper right elements of $$\Big [{\begin{array}{c}{{\varvec{\Lambda }}_{\mathbf{{s}}}} \\ {{\varvec{\Lambda }}_{\mathbf{{r}}}} \end{array}}\Big ]$$ are set to zero when $k>1$ and ${\mathbf{{D}}}$ is set to $\mathbf{{I}}_k$. Let the loadings and scores with these constraints be denoted by $$\Big [{\begin{array}{c}{{\varvec{\Lambda }}^*_{\mathbf{{s}}}} \\ {{\varvec{\Lambda }}^*_{\mathbf{{r}}}} \end{array}}\Big ]$$ and ${\mathbf{{f}}}^*$, with ${\mathbf{{f}}}^* \sim \text{ N }\big (\mathbf{{0}}, \, \mathbf{{I}}_k \otimes \mathbf{{G_g}}\big )$. The loadings and scores can be rotated back to their original form in Eq. 23 for interpretation. This rotation is given by:

29 $$\begin{aligned} \left[ \begin{array}{c}{\varvec{\Lambda }}_{\mathbf{{s}}} \\ {\varvec{\Lambda }}_{\mathbf{{r}}} \end{array}\right] = \left[ \begin{array}{c}{\varvec{\Lambda }}^*_{\mathbf{{s}}} \\ {\varvec{\Lambda }}^*_{\mathbf{{r}}} \end{array}\right] {\mathbf{{V}}}{\mathbf{{D}}}^{-1/2} \quad \text {and} \ \quad {\mathbf{{f}}} = \big ({\mathbf{{D}}}^{1/2}{{\mathbf{{V}}}}^{\scriptscriptstyle \top }\otimes \mathbf{{I}}_v\big ){\mathbf{{f}}}^*, \end{aligned}$$

where ${\mathbf{{V}}}$ is a $k\times k$ orthonormal matrix of right singular vectors and ${\mathbf{{D}}}^{1/2}$ is a $k\times k$ diagonal matrix of singular values sorted in decreasing order, with ${\mathbf{{f}}} \sim \text{ N }\big (\mathbf{{0}}, \, {\mathbf{{D}}} \otimes \mathbf{{G_g}}\big )$. These matrices can be obtained from the singular value decomposition given by:

30 $$\begin{aligned} \mathbf{{B}}\left[ \begin{array}{c}{\varvec{\Lambda }}^*_{\mathbf{{s}}} \\ {\varvec{\Lambda }}^*_{\mathbf{{r}}} \end{array}\right] = {\mathbf{{U}}}{\mathbf{{D}}}^{1/2}{{\mathbf{{V}}}}^{\scriptscriptstyle \top }\qquad \text {or} \qquad {\varvec{\Lambda }}^* = {\mathbf{{U}}}{\mathbf{{D}}}^{1/2}{{\mathbf{{V}}}}^{\scriptscriptstyle \top }, \end{aligned}$$

where ${\mathbf{{U}}}$ is a $p\times k$ orthonormal matrix of left singular vectors, with $$\Big [{\begin{array}{c}{{\varvec{\Lambda }}_{\mathbf{{s}}}} \\ {{\varvec{\Lambda }}_{\mathbf{{r}}}} \end{array}}\Big ]\equiv \mathbf{{B}}^{-1}{\mathbf{{U}}}$$ and $$\Big [{\begin{array}{c}{{\varvec{\Lambda }}^*_{\mathbf{{s}}}} \\ {{\varvec{\Lambda }}^*_{\mathbf{{r}}}} \end{array}}\Big ]\equiv \mathbf{{B}}^{-1}{\varvec{\Lambda }}^*$$, where ${\varvec{\Lambda }}^{\hskip -1pt *}$ is the loadings matrix in Eq. 10 with upper right elements set to zero (see “Appendix”). This demonstrates how the factor loadings in the IFA-LMM can be obtained directly from the fit of the conventional FA-LMM.

#### Computation

The IFA-LMM was coded in *R* (R Core Team 2021) using open source libraries. The computational approach for fitting the IFA-LMM is provided in the Supplementary Material. This approach obtains REML estimates of the variance parameters using an extension of the sparse formulation of the average information algorithm (Thompson et al. 2003). Let the REML estimates of the key variance parameters be denoted by $$\Big [{\begin{array}{c}{\hat{\varvec{\Lambda }}_{\mathbf{{s}}}} \\ {\hat{\varvec{\Lambda }}_{\mathbf{{r}}}} \end{array}}\Big ]$$ and $\hat{\varvec{\Psi }}$, with EBLUPs of the key random effects denoted by $\tilde{\mathbf{{f}}}$ and $\tilde{\varvec{\delta }}$. All linear mixed models were also fitted in *ASReml-R* (Butler 2020), with known environmental covariates included using the *mbf* argument. An example *R* script is provided in the Supplementary Material.

### Model selection

Order selection in the IFA-LMM was achieved using a combination of formal and informal criteria. Formal selection was achieved using the Akaike Information Criterion (AIC) and informal selection was achieved using two measures of variance explained. These measures are an extension of Smith et al. (2021) to include known environmental covariates, and are similar to the $R^2$ goodness-of-fit statistic in multiple regression. These measures are derived in the Supplementary Material.

The percentage of additive genetic variance explained by the known covariates and overall by the known and latent covariates is given by:

31 $$\begin{aligned} \bar{v}_s = 100\, \frac{\text {tr}\big (\mathbf{{S}}\hat{\varvec{\Lambda }}_{\mathbf{{s}}}\hat{\mathbf{{D}}}\hat{\varvec{\Lambda }}_{\mathbf{{s}}}^{{\scriptscriptstyle \top }}{\mathbf{{S}}}^{\scriptscriptstyle \top }\big )}{\text {tr}\big (\hat{\mathbf{{G}}}_{\mathbf{{e}}}\big )} \ \ \quad \text {and} \ \ \quad \bar{v} = 100\, \frac{\text {tr}\big (\hat{\mathbf{{D}}}\big )}{\text {tr}\big (\hat{\mathbf{{G}}}_{\mathbf{{e}}}\big )}, \end{aligned}$$

where $\mathbf{{G}}_{\mathbf{{e}}}$ is defined in Eq. 26. Similar measures are also obtained for the $j^{th}$ environment, that is ${v_{s_j}}$ and ${v_j}$. The final model order is typically chosen such that $\bar{v}_s$ and $\bar{v}$ are sufficiently high and the number of environments with low values of ${v_{s_j}}$ and ${v_j}$ is small. Note that this may require a different number of known and latent factors, that is $k_s$ and $k_r$.

### Model assessment

Model assessment of the IFA-LMM was achieved using the prediction accuracy for current and future environments. Prediction into *current* environments was assessed using leave-one-out cross-validation, where yield data for a single environment were excluded and then predicted. The additive GE effects for environment *j* were predicted as:

32 $$\begin{aligned} \tilde{\mathbf{{u}}}_j = \left (\big[\mathbf{{S}}_j\hat{\varvec{\Lambda }}_{\mathbf{{s}}_{-j}} + \bar{\varvec{\Lambda }}_{\mathbf{{r}}_{-j}}\big] \otimes \mathbf{{I}}_v \right ) \tilde{\mathbf{{f}}}_j, \end{aligned}$$

where $\mathbf{{S}}_j^{\scriptscriptstyle \top }$ is a *q*-vector of known covariates, $\tilde{\mathbf{{f}}}_j$ is a $v_jk$-vector of predicted scores for the $v_j$ genotypes in the $j^{th}$ current environment and $\bar{\varvec{\Lambda }}_{\mathbf{{r}}_{-j}}={\mathbf{{1}}}^{\scriptscriptstyle \top }_{p-q-1}\hat{\varvec{\Lambda }}_{\mathbf{{r}}_{-j}}/(p-1)$ ensures the scores are appropriately scaled by the latent covariates. Note that the factor loadings, $\hat{\varvec{\Lambda }}_{\mathbf{{s}}_{-j}}$ and $\hat{\varvec{\Lambda }}_{\mathbf{{r}}_{-j}}$, are estimated using data on the ($p-1$) environments excluding the $j^{th}$ environment. The prediction accuracy for environment *j* was then calculated as:

33 $$\begin{aligned} r_j = \text {cor}\big(\bar{\mathbf{{y}}}_j,\tilde{\mathbf{{u}}}_j\big), \end{aligned}$$

where $\bar{\mathbf{{y}}}_j$ is a $v_j$-vector of genotype mean yields for the $j^{th}$ current environment.

Prediction into *future* environments was assessed using a similar measure, but note that yield data for the entire year were excluded at once. The additive GE effects for environment *j* were then predicted as:

34 $$\begin{aligned} \tilde{\mathbf{{u}}}^*_j = \left (\big[\mathbf{{S}}_j^*\hat{\varvec{\Lambda }}_{\mathbf{{s}}} + \bar{\varvec{\Lambda }}_{\mathbf{{r}}}\big] \otimes \mathbf{{I}}_v \right ) \tilde{\mathbf{{f}}}^*_j, \end{aligned}$$

where $\mathbf{{S}}^{*{\scriptscriptstyle \top }}_j$ is a *q*-vector, $\tilde{\mathbf{{f}}}^*_j$ is a $v_j^*k$-vector for the $v^*_j$ genotypes in the $j^{th}$ future environment and $\bar{\varvec{\Lambda }}_{\mathbf{{r}}}={\mathbf{{1}}}^{\scriptscriptstyle \top }_{p-q}\hat{\varvec{\Lambda }}_{\mathbf{{r}}}/p$. In this case, the factor loadings, $\hat{\varvec{\Lambda }}_{\mathbf{{s}}}$ and $\hat{\varvec{\Lambda }}_{\mathbf{{r}}}$, are estimated using data on the *p* current environments only.

### Model summaries and interpretation

The main limitation of the conventional FA-LMM is that the common factors are latent so they cannot be used for interpretation or prediction. The IFA-LMM overcomes this limitation since it integrates known environmental covariates into the common factors. Interpretation is then achieved using a series of regression plots and four measures of variance explained. The regression plots are an extension of Cullis et al. (2014) and the measures of variance explained are an extension of Eq. 31.

The percentage of additive genetic variance explained by known covariate *i* is given by:

35 $$\begin{aligned} {v}_{s_i} = 100\, \frac{\big [\mathbf{{s}}_i^{\scriptscriptstyle \top }\mathbf{{S}}\hat{\varvec{\lambda }}_{\mathbf{{s}}}\hat{\mathbf{{D}}}\hat{\varvec{\lambda }}_{\mathbf{{s}}_i}^{\scriptscriptstyle \top }\big ]^2}{\big [\hat{\varvec{\lambda }}_{\mathbf{{s}}_i}\hat{\mathbf{{D}}}\hat{\varvec{\lambda }}_{\mathbf{{s}}_i}^{\scriptscriptstyle \top }\big ] \, \text {tr}\big (\hat{\mathbf{{G_e}}}\big )}, \end{aligned}$$

where $\mathbf{{G}}_{\mathbf{{e}}}$ is defined in Eq. 26. Note that $\bar{v}_s \ne \sum _{i=1}^q{v}_{s_i}$ since the known covariates are *not* orthogonal. This issue is addressed in the Supplementary Material.

The percentage of additive genetic variance explained by known factor *l* and by joint factor *l* is given by:

36 $$\begin{aligned} {v}_{s_l} = 100\, \frac{\hat{d}_l\hat{\varvec{\lambda }}_{\mathbf{{s}}_l}^{{\scriptscriptstyle \top }}{\mathbf{{S}}}^{\scriptscriptstyle \top }\mathbf{{S}}\hat{\varvec{\lambda }}_{\mathbf{{s}}_l}}{\text {tr}(\hat{\mathbf{{G}}}_{\mathbf{{e}}})} \ \ \quad \text {and} \ \ \quad {v}_l = 100\, \frac{\hat{d}_{l}}{\text {tr}(\hat{\mathbf{{G}}}_{\mathbf{{e}}})}. \end{aligned}$$

Note that $\bar{v}_s = \sum _{l=1}^k{v}_{s_l}$ and $\bar{v} = \sum _{l=1}^k{v}_{l}$ since the known and joint factors *are* orthogonal.

Lastly, the percentage of additive genetic variance in joint factor *l* explained by known covariate *i* is given by:

37 $$\begin{aligned} {v}_{li} = 100\, \left (\mathbf{{s}}_i\big [\mathbf{{S}}\hat{\varvec{\lambda }}_{\mathbf{{s}}_l} + \varvec{\Gamma }\hat{\varvec{\lambda }}_{\mathbf{{r}}_l}\big ] \right )^2. \end{aligned}$$

The percentage of variance explained by all covariates is then given by ${v}_{l\cdot } = 100\, \big[\hat{\varvec{\lambda }}_{\mathbf{{s}}_l}^{{\scriptscriptstyle \top }}{\mathbf{{S}}}^{\scriptscriptstyle \top }\mathbf{{S}}\hat{\varvec{\lambda }}_{\mathbf{{s}}_l}\big]$, which is equivalent to $v_{s_l}/v_l$ in Eq. 36.

**Table 3:** Summary of the variance models for the additive GE effects considered in this paper

Model	Description	$\mathbf{{G_e}}$		Parameters	Reference
*id*	Identity	$\sigma ^2_{ge}\mathbf{{I}}_p$		1	
*diag*	Diagonal	$\varvec{\Sigma}_\mathbf{{ge}}$	$\varvec{\Sigma}_\mathbf{{ge}}=\oplus _{j=1}^p\sigma ^2_{ge_j}$	*p*	
*comp*	Compound symmetry	$\sigma ^2_{g}\mathbf{{J}}_p + \sigma ^2_{ge}\mathbf{{I}}_p$	$\mathbf{{J}}_p=\mathbf{{1}}_p{\mathbf{{1}}}_p^{\scriptscriptstyle \top }$	2	Patterson et al. (1977)
*mdiag*	Main effects plus diagonal	$\sigma ^2_{g}\mathbf{{J}}_p + \varvec{\Sigma}_\mathbf{{ge}}$		$p+1$	Cullis et al. (1998)
FAM*k*	Factor analytic plus main effects	$\sigma ^2_{1}\mathbf{{J}}_p +\varvec{\Lambda} \mathbf{{D}}{\varvec{\Lambda }}^{\!\scriptscriptstyle \top }+\varvec{\Psi }$	$\mathbf{{D}} = \oplus _{l=1}^k d_{l}$	$p(k+1) - k(k-1)/2+1$	Smith et al. (2001)
FA*k*	Factor analytic	$\varvec{\Lambda} \mathbf{{D}}{\varvec{\Lambda }}^{\!\scriptscriptstyle \top }+\varvec{\Psi }$	$\varvec{\Psi } = \oplus _{j=1}^p\psi _{j}$	$p(k+1) - k(k-1)/2$	Smith et al. (2001)
*rreg*$_1$	Random regression 1	$\sigma ^2_{g}\mathbf{{J}}_p + \sigma ^2_s\mathbf{{S}}{\mathbf{{S}}}^{\scriptscriptstyle \top }+\varvec{\Psi }$		$p+2$	Jarquín et al. (2014)
*rreg*$_2$	Random regression 2	$\sigma ^2_{g}\mathbf{{J}}_p + \mathbf{{S}} \varvec{\Sigma} _\mathbf{{s}}{\mathbf{{S}}}^{\scriptscriptstyle \top }+\varvec{\Psi }$	$\varvec{\Sigma}_\mathbf{{s}}=\oplus _{i=1}^q\sigma ^2_{s_i}$	$p+q+1$	Heslot et al. (2014)
FAR*k*	Factor analytic regression	$[\mathbf{{1}}^\star _p \ \mathbf{{S}}]\varvec{\Lambda}_\mathbf{{a}} \mathbf{{D}}{\varvec{\Lambda }}_{\mathbf{{a}}}^{\scriptscriptstyle \top }[\mathbf{{1}}^\star _p \ \mathbf{{S}}{]}^{\scriptscriptstyle \top }+\varvec{\Psi }$	$$\varvec{\Lambda}_\mathbf{{a}}=\Big [{\begin{array}{c}{{\varvec{\Lambda }}_{\mathbf{{g}}}} \\ {{\varvec{\Lambda }}_{\mathbf{{s}}}} \end{array}}\Big ]$$	$p+ k(2q-k+3)/2$	Jennrich and Schluchter (1986)
IFA*k*	Integrated factor analytic	$[\mathbf{{S}} \ \varvec{\Gamma }]\varvec{\Lambda}_\mathbf{{b}} \mathbf{{D}}{\varvec{\Lambda }}_{\mathbf{{b}}}^{\scriptscriptstyle \top }[\mathbf{{S}} \ \varvec{\Gamma }{]}^{\scriptscriptstyle\top }+\varvec{\Psi }$	$$\varvec{\Lambda}_\mathbf{{b}}=\Big [{\begin{array}{c}{{\varvec{\Lambda }}_{\mathbf{{s}}}} \\ {{\varvec{\Lambda }}_{\mathbf{{r}}}} \end{array}}\Big ]$$	$p(k+1) - k(k-1)/2$	This paper

Presented for each model is the structure of the additive genetic variance matrix between environments ($\mathbf{{G_e}}$), number of estimated variance parameters and the reference

*Note:* The *vp*-vector of additive GE effects is given by $\mathbf{{u}}$ with var$(\mathbf{{u}})=\mathbf{{G_e}}\otimes \mathbf{{G_g}}$, where $\mathbf{{G}}_{\mathbf{{e}}}^{p\times p}$ is the variance matrix between environments and $\mathbf{{G}}_{\mathbf{{g}}}^{v\times v}$ is the genomic relationship matrix between genotypes. Also note that $\mathbf{{1}}^\star _p=\mathbf{{1}}_p/\sqrt{p}$, $\varvec{\Lambda }^{p\times k}$ is a matrix of latent covariates with *p* environments and *k* factors, $\mathbf{{S}}^{p\times q}$ is a matrix of known covariates with *q* covariates and $\varvec{\Gamma }^{p\times (p-q)}$ is an orthogonal projection matrix, with ${\mathbf{{S}}}^{\scriptscriptstyle \top }\varvec{\Gamma }=\mathbf{{0}}$

**Table 4:** Linear mixed models with random regressions on latent environmental covariates

Regressions on latent covariates											
**(a)** Models with simple main effects						**(b)** Models with generalised main effects$^*$					
Model	Pars	Loglik	AIC	${v} _g$	$\bar{v}$	Model	Pars	Loglik	AIC	${v}_1$	$\bar{v}$
*comp*	2	10,504.2	− 20,748.4	36.2	36.2	*id*	1	10,156.9	− 20,055.9	$-$	$-$
*mdiag*	25	10,563.6	− 20,821.1	33.6	33.6	*diag*	24	10,249.3	− 20,194.7	–	–
FAM1	49	10,765.4	− 21,176.8	36.8	54.4	FA1	48	10,667.1	− 20,982.2	43.2	43.2
FAM2	72	10,893.8	− 21,387.6	37.2	67.5	FA2	71	10,827.4	− 21,256.8	44.1	60.4
FAM3	94	10,942.9	− 21,441.8	38.2	72.0	FA3	93	10,940.3	− 21,438.5	43.8	70.7
**FAM4**	**115**	**10,981.7**	**− 21,477.5**	**38.1**	**76.9**	**FA4**	**114**	**10,978.3**	**− 21,472.5**	**43.8**	**75.2**
FAM5	135	11,011.2	− 21,496.5	38.7	80.0	FA5	134	11,010.1	− 21,496.1	44.3	79.0

Presented for each model is the number of estimated genetic variance parameters, residual log-likelihood, AIC and percentage of variance explained by the simple (${v}_g$) or generalised (${v}_1$) main effects and overall ($\bar{v}$)

*Note:* 128 non-genetic and residual variance parameters estimated in all models. The selected FAM4 and FA4 models are distinguished with *bold font*

^*^Models where intercepts are not explicitly fitted

## Results

This section presents the results of model fitting using the 2017 P1 MET dataset and model assessment using the 2018 P2 MET dataset. The P1 dataset is summarised in Tables 1 and 2, and comprises $v=204$ genotypes evaluated in $p=24$ current environments with $q=18$ known covariates. The P2 dataset is summarised in Supplementary Tables 9 and 10, and comprises $v^*=55$ (of the 204) genotypes evaluated in $p^*=20$ future environments with the same known covariates. The results are presented according to model selection, assessment and interpretation.

### Model selection

Tables 4 and 5 present the model selection criteria previously described in “Model selection”. The important results from each model fit are detailed below.

#### Baseline linear mixed models

The analyses commenced by fitting a linear mixed model with a diagonal model for the additive GE effects (*diag*; Table 4b). This approach reflects the initial single-site analyses routinely performed on MET datasets, where the additive GE effects in different environments are assumed to be independent. The single-site analyses are typically used to inspect the experimental design, address spatial variations and identify potential outliers.

The analyses continued by fitting a linear mixed model with a compound symmetry model for the additive GE effects (*comp*; Table 4a). This approach reflects many current applications of GS in plant breeding, where the additive GE effects in different environments are assumed to be correlated. The compound symmetry model is very restrictive, however, since it comprises a single variance component for the simple genotype main effects and genotype by environment interaction effects. This model can be extended to include heterogeneous interaction variances across environments, that is the main effects plus diagonal model (*mdiag*; Table 4a). The AIC for this model is much lower, and thence much better, than the standard compound symmetry model. There are negligible differences between the overall additive genetic variance explained, however, with $\bar{v} \approx 35\%$ for both models.

#### Regressions on latent covariates

A series of factor analytic linear mixed models were then fitted with either (a) simple or (b) generalised main effects (Table 4). The most notable differences between the FAM-LMMs and FA-LMMs are observed in the lower orders, where the overall additive genetic variance explained by the latent common factors is low. At the higher orders, where the overall variance explained is sufficiently high, the differences are negligible. Both models required $k=4$ latent factors to reach a sufficient percentage of additive genetic variance explained for individual environments and overall, with ${v_j}>40$% and $\bar{v}>75\%$. Lastly, note that the generalised main effects in (b) explain 5.7% more variance than the simple main effects in (a), despite very similar overall variance explained. This feature is now discussed.

**Fig. 2:**
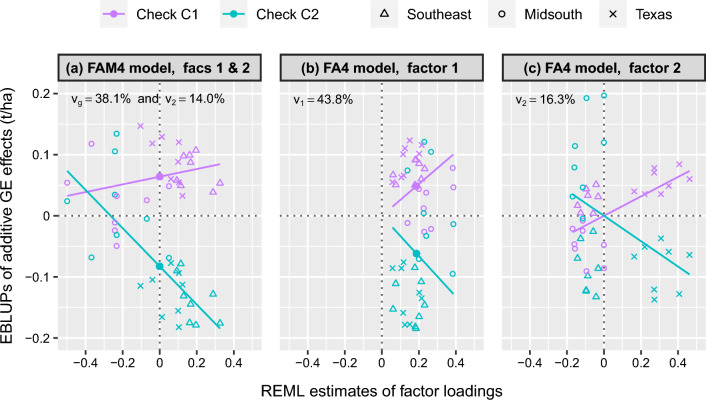
Regression plots for checks C1 and C2 in terms of the first two factors obtained from the **a** FAM4 and **b/c** FA4 models. *Note:* The simple main effects in **a** and the generalised main effects in **b** are denoted with *closed circles* and the growing regions are distinguished by *shape*. The percentage of additive genetic variance explained by each factor is labelled. The additive GE effects in **c** have been adjusted for those in **b**

The simple and generalised main effects are demonstrated in Fig. 2. This figure presents a series of regression plots for checks C1 and C2 in terms of the (a) FAM4 and (b/c) FA4 models. Recall that the FAM4 model can be viewed as a special FA5 model where the first factor loadings are equal and correspond to the simple main effects, whereas the higher order loadings sum to zero and correspond to the interaction effects. The first two factors are plotted for the FAM4 model in Fig. 2a where the simple main effects are denoted by the fitted values of the *second* factor regressions at the mean loading of zero, that is 0.06 and − 0.09 t/ha for C1 and C2. In contrast, the generalised main effects for the FA4 model in Fig. 2b are denoted by the fitted values of the *first* factor regressions at the mean loading of 0.19, that is 0.05 and − 0.06 t/ha. There are two important differences between these approaches:

1. The generalised main effects capture heterogeneity of scale variance, that is non-crossover GEI, whereas the simple main effects do not capture GEI. This is demonstrated in Fig. 2b where the regression lines diverge across environments so the genotype rankings never crossover, whereas the first factor regression lines in the FAM4 model are always parallel (not shown).
2. The higher order factors in the FA4 model predominately capture crossover GEI only, whereas those in the FAM4 model capture some mixture of non-crossover and crossover GEI. This is demonstrated in Fig. 2c where the regression lines intersect so the genotype rankings crossover, whereas the regression lines in Fig. 2a diverge as well as crossover.

#### Regressions on known covariates

The next two linear mixed models fitted include random regressions without translational invariance. The random regression in Jarquín et al. (2014) reflects a popular application of GS in plant breeding (*rreg*$_1$; Table 5a). Like the compound symmetry model, however, this model is very restrictive since it only comprises two variance components. The only difference is that the interaction effects are now parametrised by known environmental covariates. This model can be extended to include heterogeneous interaction variances across covariates (*rreg*$_2$; Table 5a). The AIC for the random regression in Heslot et al. (2014) is much better than the simpler random regression. There are negligible differences between the additive genetic variance explained, however, with $\bar{v}_s\approx 23\%$ and $\bar{v}\approx 58\%$ for both models. Interestingly, the former measure matches that reported in Jarquín et al. (2014).

A series of FAR-LMMs with translational invariance were then fitted (Table 5a). This approach required $k=4$ known factors to reach a sufficient percentage of additive genetic variance explained for individual environments and overall, with $v_{j}>40\%$ and $\bar{v}= 70.7$%. The AIC for the FAR4 model is substantially better than the random regressions in Jarquín et al. (2014) and Heslot et al. (2014). The FAR4 model also explains more additive genetic variance in the known covariates, with $\bar{v}_s=33.2\%$ compared to only 20.8 and 23.2 %. This demonstrates the importance of appropriately modelling the variance structure between known covariates.

**Table 5:** Linear mixed models with random regressions on known and latent environmental covariates

Regressions on known covariates						Regressions on known and latent covariates					
**(a)** Models with simple main effects						**(b)** Models with generalised main effects^*^					
Model	Pars	Loglik	AIC	$\bar{v}_s$	$\bar{v}$	Model	Pars	Loglik	AIC	$\bar{v}_{s}$	$\bar{v}$
*rreg*$_1$	26	10,721.2	− 21,134.3	20.8	57.1	*id*	1	10,156.9	− 20,055.9	$-$	$-$
*rreg*$_2$	43	10,750.7	− 21,159.3	23.2	58.5	*diag*	24	10,249.3	− 20,194.7	$-$	$-$
FAR1	43	10,636.7	− 20,931.4	6.2	40.0	IFA1	48	10,667.1	− 20,982.2	7.0	43.2
FAR2	61	10,791.4	− 21,204.8	19.2	57.0	IFA2	71	10,827.4	− 21,256.8	20.1	60.4
FAR3	78	10,887.0	− 21,361.9	29.2	66.7	IFA3	93	10,940.3	− 21,438.5	30.1	70.7
**FAR4**	**94**	**10,911.7**	**− 21,379.4**	**33.2**	**70.7**	**IFA4-3**	**108**	**10,971.9**	**− 21,471.9**	**34.4**	**74.9**
FAR5	109	10,931.3	− 21,388.7	36.2	73.8	IFA5-3	122	10,996.4	− 21,492.8	36.2	78.0

Presented for each model is the number of estimated genetic variance parameters, residual log-likelihood, AIC and percentage of variance explained by the known covariates ($\bar{v}_{s}$) and overall ($\bar{v}$)

*Note:* 128 non-genetic and residual variance parameters estimated in all models. The models *rreg*$_1$ and *rreg*$_2$ correspond to the random regressions in Jarquín et al. (2014) and Heslot et al. (2014). The selected FAR4 and IFA4-3 models are distinguished with *bold font*.

^*^Models where intercepts are not explicitly fitted

#### Regressions on known and latent covariates

The analyses concluded by fitting a series of IFA-LMMs with generalised main effects and translational invariance (Table 5b). This approach required $k_s=4$ known and $k_r=3$ latent factors to reach a sufficient percentage of additive genetic variance explained for individual environments and overall, with $v_{j}>45\%$ and $\bar{v}= 74.9\%$. The AIC for the IFA4-3 model is substantially better than the FAR4 model. The IFA4-3 model also explains more overall variance, that is $\bar{v}=74.9\%$ compared to 70.7%, despite similar variance explained by the known covariates, with $\bar{v}_s\approx 35\%$ for both models. This demonstrates the advantage of including generalised main effects based on latent environmental covariates, instead of simple main effects.

### Model comparison

The IFA4-3 model provides a good fit to the MET dataset and captures a large proportion of additive genetic variance (Table 5). The FAM4 and FA4 models also provide a good fit and capture a large proportion of variance, but they cannot be used for prediction into future environments (Table 4). The random regression models in Jarquín et al. (2014) and Heslot et al. (2014) can be used for prediction, but they provide a poor fit, capture the lowest variance of all models and are not translational invariant. The FAR4 model provides a better fit and captures more variance than the simpler random regression models, and is translational invariant. The IFA4-3 model provides an even better fit, captures more variance than the FAR4 model and is also translational invariant; making it the preferred method of analysis in this paper.

### Model assessment

The mean prediction accuracy of the IFA4-3 model is considerably higher than all other random regression models (Table 6). The prediction accuracy was calculated in terms of 24 current environments in 2017 P1 and 20 future environments in 2018 P2. The most notable differences between models are observed for the 2018 environments in Texas, where the accuracy of the IFA4-3 model is at least 0.22 higher. In the Southeast and Midsouth, the accuracies are at least 0.06 and 0.10 higher, respectively. The differences in Texas are negligible for the 2017 environments, where the accuracies are generally higher for all models. In the Southeast and Midsouth, however, the accuracies of the IFA4-3 model are still at least 0.09 higher.

**Table 6:** Summary of the prediction accuracies for the 2017 current and 2018 future environments

Year	Model	$\scriptstyle \triangle$ Southeast			° Midsouth			$\times$ Texas			Overall		
		Min	Mean	Max	Min	Mean	Max	Min	Mean	Max	Min	Mean	Max
	*rreg*$_1$	0.27	0.51	0.68	0.30	0.58	0.77	0.27	0.47	0.60	0.27	0.52	0.77
	*rreg*$_2$	0.27	0.52	0.69	0.29	0.58	0.76	0.27	0.47	0.61	0.27	0.52	0.76
	FAR4	0.25	0.50	0.66	0.34	0.59	0.77	0.25	0.48	0.64	0.25	0.52	0.77
2017	**IFA4-3**	**0.33**	**0.60**	**0.76**	**0.45**	**0.68**	**0.79**	**0.29**	**0.50**	**0.65**	**0.29**	**0.60**	**0.79**
	*rreg*$_1$	0.58	0.60	0.64	0.30	0.50	0.71	− 0.03	0.20	0.34	− 0.03	0.42	0.71
	*rreg*$_2$	0.58	0.61	0.64	0.28	0.49	0.70	− 0.02	0.21	0.36	− 0.02	0.42	0.70
	FAR4	0.58	0.61	0.67	0.26	0.49	0.71	0.02	0.22	0.36	0.02	0.43	0.71
2018	**IFA4-3**	**0.60**	**0.67**	**0.71**	**0.31**	**0.60**	**0.79**	**0.30**	**0.44**	**0.62**	**0.30**	**0.56**	**0.79**

Presented for each model is the minimum, mean and maximum prediction accuracy for the $\scriptstyle \triangle$ Southeast, ° Midsouth and $\times$ Texas, as well as overall across all regions

*Note:* The models *rreg*$_1$ and *rreg*$_2$ correspond to the random regressions in Jarquín et al. (2014) and Heslot et al. (2014). The highest accuracy is distinguished with *bold font*

### Model summaries and interpretation

**Fig. 3:**
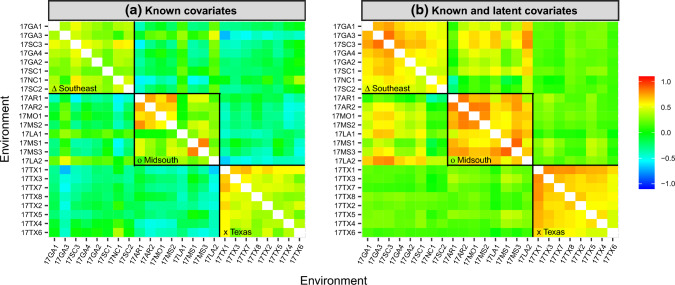
Heatmaps of the additive genetic correlation matrices between environments in terms of the **a** known covariates and **b** known and latent covariates. *Note*: Both matrices are ordered using the dendrogram applied to **b**. *Black lines* distinguish the $\scriptstyle \triangle$ Southeast, ° Midsouth and $\times$ Texas cotton growing regions. The colourkey ranges from 1 (agreement in rankings) through zero (dissimilarity in rankings) to −1 (reversal of rankings)

**Fig. 4:**
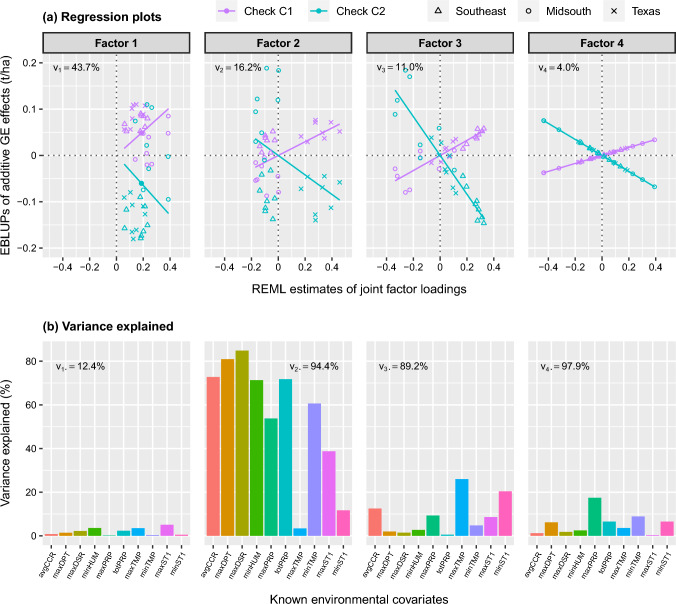
**a** Regression plots for checks C1 and C2 in terms of four joint factors and **b** percentage of additive genetic variance in the joint factors explained by the known covariates. *Note*: The generalised main effects in **a** are denoted with *closed circles* and the growing regions are distinguished by *shape*. The percentage of variance explained by each factor is labelled in **a** and the percentage of variance in each factor explained by all known covariates is labelled in **b**. The additive GE effects for the higher order factors are adjusted for the preceding factor(s). Only 10 (of the 18) known covariates are displayed in **b** for brevity

Tables 7, 8 and Figs. 3, 4 present the model summaries previously described in  “Model summaries and interpretation”. These summaries are presented for the IFA4-3 model in terms of environments, covariates and genotypes.

#### Summary of environments and covariates

Table 7 presents a summary of the growing environments in the 2017 P1 MET dataset. The additive genetic variance of individual environments range from 0.01 to 0.06, with mean of 0.03. These variances are obtained from the diagonal elements of the denominator in Eq. 31. The overall variance explained by the known and latent covariates is much higher than the variance explained by the known covariates alone, that is ${v_{j}} = 44.3 - 100.0$ % with $\bar{v} =74.9\%$ compared to ${v_{s_j}} = 12.5-85.4$ % with $\bar{v}_s =34.4\%$. Most variance is explained overall in the Midsouth (84.9% compared to only 66.6 and 69.3%), whereas most variance is explained by the known covariates in Texas (41.1% compared to only 28.4 and 33.4 %). Table 7 also presents REML estimates of the joint factor loadings. The first factor comprises positive loadings only, and explains ${v}_{1}=43.7$% of the additive genetic variance. The higher order factors comprise both positive and negative loadings, and explain ${v}_{l}= 4.0-16.2$ %, with 31.2% in total. The sign of the loadings indicate that the first factor captures non-crossover GEI only, whereas the higher order factors predominately capture crossover GEI only (Smith and Cullis 2018).

**Table 7:** The selected IFA4-3 model, Part 1: Summary of growing environments

State	Env	Var	${v}_{{s}_j}$	${v_j}$	$\hat{\varvec{\lambda }}_{1}$	$\hat{\varvec{\lambda }}_{2}$	$\hat{\varvec{\lambda }}_{3}$	$\hat{\varvec{\lambda }}_{4}$
$\scriptstyle \triangle$ North Carolina	17NC1	0.01	85.4	69.3	0.06	− 0.04	0.33	0.06
	17SC1	0.02	12.5	56.4	0.18	− 0.06	0.17	− 0.15
	17SC2	0.01	40.7	48.6	0.07	− 0.03	0.27	− 0.09
$\scriptstyle \triangle$ South Carolina	17SC3	0.02	23.8	90.5	0.23	− 0.14	0.26	−0.03
	17GA1	0.03	23.1	63.8	0.20	− 0.08	0.29	− 0.02
	17GA2	0.03	19.1	54.0	0.19	− 0.10	0.31	0.01
	17GA3	0.02	29.8	82.3	0.21	− 0.12	0.20	− 0.09
$\scriptstyle \triangle$ Georgia	17GA4	0.02	26.9	67.6	0.18	− 0.10	0.28	0.14
° Missouri	17MO1	0.06	26.6	82.2	0.39	− 0.17	− 0.15	0.39
	17AR1	0.01	49.1	100.0	0.14	0.00	− 0.32	0.09
° Arkansas	17AR2	0.06	32.1	89.2	0.39	− 0.16	− 0.34	0.30
	17MS1	0.03	46.0	81.6	0.23	0.00	− 0.26	− 0.44
	17MS2	0.03	47.5	77.6	0.24	− 0.12	− 0.15	0.23
° Mississippi	17MS3	0.03	37.3	100.0	0.26	− 0.09	− 0.23	− 0.43
	17LA1	0.03	19.9	71.8	0.23	− 0.17	− 0.01	− 0.32
° Louisiana	17LA2	0.02	22.5	76.4	0.20	− 0.10	0.11	− 0.07
	17TX1	0.02	61.4	91.8	0.15	0.39	0.04	0.09
	17TX2	0.02	36.6	61.9	0.12	0.28	0.10	0.17
	17TX3	0.05	41.5	74.0	0.21	0.46	0.07	− 0.06
	17TX4	0.01	32.6	64.6	0.10	0.22	0.07	− 0.17
	17TX5	0.04	29.9	62.0	0.20	0.34	0.01	− 0.18
	17TX6	0.01	80.7	44.3	0.06	0.17	0.05	0.10
	17TX7	0.02	44.4	66.5	0.12	0.33	− 0.01	0.02
$\times$ Texas	17TX8	0.02	24.1	72.0	0.13	0.28	0.12	0.19
**Overall**	–	**0.03**	**34.4**	**74.9**	**43.7**	**16.2**	**11.0**	**4.0**

Presented are the REML estimates of additive genetic variance, percentage of variance explained by the known covariates (${v}_{{s}_j}$) and overall (${v_j}$), as well as estimates of the joint factor loadings ($\hat{\varvec{\lambda }}_{l}$)

*Note:* The percentage of variance explained across all environments ($\bar{v}_{s}$ and $\bar{v}$), as well as by individual factors (${v}_{l}$) is presented in the final row. The measure ${v_{s_j}}$ is greater than ${v_{j}}$ for 17NC1 and 17TX6 since the known and latent covariates are not orthogonal for individual environments

Table 8 presents a similar summary for the known environmental covariates in the MET dataset. The additive genetic covariance of individual covariates range from − 0.33 to 0.25, with mean of 0.01. These covariances are obtained from the square-root of the elements in Eq. 37. The variance explained by individual covariates is ${v}_{s_i}= 0.1-10.1$ %, with $\bar{v}_s= 34.4$%. The most notable covariates are maxDSR (10.1%), avgCCR (4.5%) and maxTMP (4.0%). Table 8 also presents REML estimates of the known factor loadings. The interpretation of these loadings is similar to above, but note that the higher order factors explain more additive genetic variance than the first factor, with 29.0% in total compared to only 5.4%. This will be discussed further below.

**Table 8:** The selected IFA4-3 model, Part 2: Summary of known environmental covariates

Covariate	Covar	${v}_{{s}_i}$	$\hat{\varvec{\lambda }}_{\mathbf{{s}}_1}$	$\hat{\varvec{\lambda }}_{\mathbf{{s}}_2}$	$\hat{\varvec{\lambda }}_{\mathbf{{s}}_3}$	$\hat{\varvec{\lambda }}_{\mathbf{{s}}_4}$
LAT	0.02	0.4	0.02	0.10	− 0.21	− 0.20
LONG	0.05	0.5	− 0.18	0.04	0.56	0.33
avgCCR	− 0.18	4.5	− 0.37	0.31	− 0.02	0.29
maxDPT	0.25	3.7	0.47	− 0.46	− 0.68	− 0.22
maxDSR	0.25	10.1	− 0.30	0.41	− 0.10	0.17
minHUM	− 0.33	3.5	− 0.62	0.24	1.03	1.10
maxNSR	0.04	1.9	0.05	0.11	− 0.19	− 0.29
maxPRP	− 0.01	0.1	0.04	0.05	− 0.18	− 0.55
totPRP	0.03	1.6	0.11	− 0.01	0.05	− 0.15
maxTMP	0.18	4.0	− 0.31	0.09	0.58	0.32
minTMP	− 0.18	3.1	− 0.05	0.44	− 0.67	− 1.00
minWSP	0.01	0.1	− 0.13	− 0.09	0.31	0.16
avgWDR	− 0.03	1.5	0.03	0.14	− 0.01	− 0.33
maxST1	− 0.04	1.0	0.09	0.06	− 0.27	− 0.25
minST1	0.04	0.1	0.37	− 0.48	0.15	0.96
avgSM3	− 0.02	0.4	0.10	0.12	0.10	0.19
avgSM4	0.05	1.2	− 0.10	− 0.15	− 0.25	− 0.41
minST4	0.09	1.4	− 0.30	0.32	0.10	− 0.48
**Overall**	**0.01**	**34.4**	**5.4**	**15.3**	**9.8**	**3.9**

Presented are the REML estimates of additive genetic covariance, percentage of variance explained by individual known covariates (${v}_{{s}_i}$) and estimates of the known factor loadings ($\hat{\varvec{\lambda }}_{\mathbf{{s}}_l}$)

*Note:* The percentage of variance explained by all known covariates ($\bar{v}_{s}$) and by individual factors (${v}_{s_l}$) is presented in the final row

#### Correlations between environments

Figure 3 presents heatmaps of the additive genetic correlation matrices between environments in terms of the (a) known covariates and (b) known and latent covariates. These matrices are ordered based on the dendrogram constructed using the *agnes* function in the *cluster* package (Maechler et al. 2019). This dendrogram generally places environments closer together that have more similar GEI patterns than those further apart. Figure 3 suggests there is structure to the GEI underlying the heatmaps. There are three notable features:

1. The overall correlations based on the known and latent covariates are considerably higher than the correlations based on the known covariates alone.
2. The highest overall correlations generally occur between environments in the same growing region. Environments in the Southeast and Midsouth are also well correlated.
3. The overall correlations between environments in the same growing region are less than one. This indicates that crossover GEI is present within regions.

#### Regression plots for genotypes

Figure 4a presents a series of regression plots for checks C1 and C2 in terms of the $k=4$ joint factors in the IFA4-3 model. These plots are used to assess genotype performance and stability in response to the known and latent environmental covariates. These plots show that check C1 is generally higher performing than C2 since it has a higher predicted slope for the first factor regression, that is 0.26 compared to − 0.32. Both checks are considerably unstable, however, since they have large slopes for the higher order factors and therefore have large deviations about the first factor regression. Figure 4a also suggests that the second factor is correlated with longitude (Pearson's $r = 0.80$), where the loadings on the left correspond to the Southeast and Midsouth while the loadings on the right correspond to Texas. This highlights an important limitation of the conventional FA-LMM, where interpretation is often limited to post-processing of the latent factors. This will be discussed further below.

Figure 4b presents direct interpretation of the factors in terms of the variance explained by the known environmental covariates. This figure suggests there is structure to the GEI underlying the regression plots. There are three notable features:

1. The known covariates predominately model crossover GEI, with ${v}_{{l\cdot }}=89.2-97.9$ % of the additive genetic variance explained in the higher order factors compared to only ${v}_{{1\cdot }}=12.4\%$ explained in the first factor. These measures are obtained from Eq. 36, and are equivalent to ${v_{s_l}}/{v_l}$ in Tables 7 and 8.
2. The second factor is well explained by multiple known covariates. This demonstrates the biological drivers of crossover GEI in this factor, that is the drivers of crossover GEI due to changes in LONG.
3. The third and fourth factors are not well explained by individual covariates. This indicates that crossover GEI in these factors is driven by a combination of known covariates as well as their interaction.

## Discussion

This paper developed a single-stage GS approach which integrates known and latent environmental covariates within a special factor analytic framework. The FA-LMM of Smith et al. (2001) is an effective method for analysing MET datasets, but has limited practicality since the underlying factors are latent so the modelled GEI is observable, rather than predictable. The advantage of using random regressions on known environmental covariates is that the modelled GEI becomes predictable. The IFA-LMM developed in this paper includes a model for predictable and observable GEI in terms of a joint set of known and latent environmental covariates.

Regressions on known environmental covariates were first used in plant breeding by Yates and Cochran (1938). Their work was later popularised by Finlay and Wilkinson (1963), and includes a fixed coefficient regression on a set of environmental mean yields (covariates). Despite its popularity, however, there is a fundamental problem with using mean yields as covariates (Knight 1970; Freeman and Perkins 1971). This problem can be overcome by implementing environmental covariates which are independent of the genotypes under study, such as soil moisture and daily temperature (Hardwick and Wood 1972; Fripp 1972). Several authors have also used fixed regressions on genotype covariates, such as disease resistance and maturity, in addition to the environmental covariates. This approach is often referred to as fixed factorial regression (Denis 1980, 1988).

An alternative approach is to use a linear mixed model with a random coefficient regression. This approach was popularised by Laird and Ware (1982), and requires an appropriate variance model for the intercepts and slopes which ensures the regression is scale and translational invariant. An appropriate choice is the fully unstructured variance model, however, this model becomes computationally prohibitive as the number of covariates increases. Recently, Heslot et al. (2014) extended the random regression model for GS, but they were unable to fit an appropriate variance model (also see Jarquín et al. 2014). The FAR-LMM developed in this paper includes a reduced rank factor analytic variance model for the intercepts and slopes. This ensures the regression is computationally efficient as well as both scale and translational invariant, regardless of the number of covariates. The selected FAR-LMM also provides a substantially better fit and captures more additive genetic variance than the simpler random regression models.

The FAR-LMM includes a set of *simple* main effects which reflect simple averages across environments. Smith and Cullis (2018) discuss the limitations of simple main effects, and demonstrate how *generalised* main effects can be obtained from FA-LMMs. They also discuss how the generalised main effects capture heterogeneity of scale variance, that is non-crossover GEI, whereas the simple main effects do not. The generalised main effects can therefore be viewed as weighted averages across environments which are based on differences in scale variance. This highlights an important difference to the simple main effects, which are more restrictive and based on a single genetic variance across environments. This feature is demonstrated in Fig. 2 for the FA-LMM and the FAM-LMM, where the generalised main effects capture $\sim 6\%$ more additive genetic variance than the simple main effects.

The IFA-LMM is an effective method for analysing MET datasets which also utilises crossover and non-crossover GEI for genomic prediction into current and future environments. The IFA-LMM is effective since it exploits the desirable features of the FAR-LMM and the FA-LMM. That is, it exploits the ability of random regression models to capture crossover GEI for prediction using known covariates and the ability of factor analytic models to capture non-crossover GEI using latent covariates. The IFA-LMM can therefore be viewed as a random factorial regression, with known genotype covariates derived from marker data, known environmental covariates derived from weather and soil data as well as latent environmental covariates estimated from the phenotypic data itself. The IFA-LMM can also be viewed as a linear mixed model analogue to redundancy analysis (Van Den Wollenberg 1977), where the factors are constrained to be linear combinations of known and latent environmental covariates. The selected IFA-LMM provides a substantially better fit and captures more additive genetic variance than the selected FAR-LMM and the simpler random regression models.

There are three appealing features of the IFA-LMM which address several long-standing objectives of many plant breeding programmes:

1. The IFA-LMM includes a regression model for GEI in terms of a small number of known and latent factors. This simultaneously reduces the dimension of the known and latent environmental covariates.
2. The regression model captures *predictable* GEI in terms of *known* environmental covariates. This is predominately in the form of crossover GEI, and enables meaningful interpretation and prediction into any current or future environment.
3. The regression model also captures *observable* GEI in terms of *latent* environmental covariates, which are orthogonal to the known covariates. This is predominately in the form of non-crossover GEI, and enables a large proportion of GEI to be captured by the regression model overall.

The IFA-LMM was demonstrated on a late-stage cotton breeding MET dataset. This dataset is an example of a small *in situ* training population which comprises a subset of current test genotypes and growing environments in 2017. A larger MET dataset across multiple years and locations is required, however, to capture the extent of transient and static GEI in the cotton growing regions of USA. This will ensure the scope of the known and latent covariates are relevant for prediction into future environments. Computational challenges are anticipated for these larger MET datasets and finding efficient ways to scale the IFA-LMM is the topic of current research.

There are four important points from “Results”:

1. The IFA4-3 model has fewer genetic variance parameters compared to the FA4 and FAM4 models, despite very similar model selection criteria (Tables 4 and 5). This highlights an important advantage of implementing known environmental information into the common factors. The IFA4-3 model also has better selection criteria than the FAR4 model. This also highlights the advantage of implementing generalised main effects based on latent environmental covariates, instead of simple main effects.
2. The known environmental covariates explain $\bar{v}_s=34.4$% of the overall additive genetic variance, which represents 93.0% of the crossover GEI captured by the regression model. This is at least 11% more variance compared to the random regression models in Jarquín et al. (2014) and Heslot et al. (2014).
3. The latent environmental covariates explain 40.5% of the overall additive genetic variance, which represents 87.6% of the non-crossover GEI. This feature can be visualised in Fig. 3 where the overall correlations based on the known and latent covariates are much higher than those based on the known covariates alone.
4. The mean prediction accuracy of the IFA4-3 model is 0.02–0.10 higher than all other random regression models for *current* environments and $0.06-0.24$ higher for *future* environments (Table 6). This highlights another important advantage of implementing known environmental information into the common factors.

Point 4 is now discussed further. The mean prediction accuracy of the IFA4-3 model was considerably higher than all other random regression models, especially for future environments in Texas. The prediction accuracy was calculated in terms of 24 current environments in 2017 P1 and 20 future environments in 2018 P2 (Table 6). The accuracy of all models were generally low for Texas in 2018, with mean of $0.20-0.44$ for all models. This suggests that GEI is more complex in Texas and that there is substantial transient GEI present across years in addition to static GEI across locations (Cullis et al. 2000). It also suggests that the crossover GEI captured by the known covariates may not be repeatable across years and that the generalised main effects based on the latent covariates may not accurately capture the true non-crossover GEI across years. That is, the current scope of the known and latent covariates is less relevant for Texas compared to the Southeast and Midsouth. The application of a larger multi-year MET dataset should overcome these issues.

Another key feature of the IFA-LMM is the ability to identify the biological drivers of GEI, such as maximum downward solar radiation and average cloud cover. Interpretation within the IFA-LMM was demonstrated using a series of regression plots (Fig. 4). These plots are used to assess genotype performance and stability in response to the known and latent environmental covariates. Previously, interpretation within factor analytic linear mixed models was limited to post-processing of model terms, for example by correlating known covariates with latent factors (Oliveira et al. 2020) or by examining the response of reference genotypes in different environments (Mathews et al. 2011). The distinguishing feature of the IFA-LMM is the ability to ascribe direct biological interpretation to the modelled GEI. This feature has three important practical implications:

1. The first factor captures non-crossover GEI only, and is predominately explained by the latent environmental covariates. The higher order factors capture crossover GEI, and are predominately explained by the known environmental covariates. This enables the drivers of GEI across a set of target environments to be identified.
2. The importance of known covariates as drivers of GEI can be quantified. This provides information on which covariates should be measured with high accuracy, say, and which covariates may be less important or don’t need to be measured at all. This is particularly appealing with the advent of high-throughput environmental data.
3. Genomic selection tools can be applied to obtain measures of overall performance and stability for each genotype. This will enable the drivers of genotype performance and stability across a set of target environments to be identified. This is the topic of a subsequent paper.

The IFA-LMM is an effective method for analysing MET datasets which also utilises crossover and non-crossover GEI for genomic prediction into current and future environments. This is becoming increasingly important with the emergence of rapidly changing environments and climate change.

### Supplementary Information

Below is the link to the electronic supplementary material.

Supplementary file 1 (PDF 496 KB)

Supplementary file 2 (TXT 13 KB)

## Data Availability

The data that support the findings of this study are available from Bayer CropScience. Restrictions apply to the availability of these data, which were used under license for this study.

## Appendix: Orthogonal matrix rotations

This appendix demonstrates how simple or generalised main effects can be obtained from factor analytic models regardless of whether intercepts are explicitly fitted. The simple main effects require rotation of the loadings and scores using a Gram-Schmidt process, whereas the generalised main effects require rotation to a principal component solution. The two rotations are detailed below.

### Gram-Schmidt process

Smith (1999) discuss the need to column centre the environmental loadings in the FAM*k* model so they are orthogonal to the simple main effects. This is achieved using a Gram-Schmidt process, with:

$$\begin{aligned}{}[\mathbf{{1}}_p^\star \ \varvec{\Lambda }^{\hskip -1pt\star} ] = [\mathbf{{1}}_p^\star \ \varvec{\Lambda }]\mathbf{{U}}^{-1} \quad \text {and} \quad \left( \begin{array}{c} \varvec{\gamma }_{\mathbf{{g}}} \\ \, \mathbf{{f}}^\star \end{array}\right) = \big (\mathbf{{U}} \otimes \mathbf{{I}}_v\big ) \left( \begin{array}{c} \varvec{\gamma }_{\mathbf{{1}}} \\ \mathbf{{f}} \end{array}\right) , \end{aligned}$$

with ${\varvec{\Lambda }}^{\hskip -1pt \star {\scriptscriptstyle \top }}\mathbf{{1}}_p^\star =\mathbf{{0}}$, where $\mathbf{{1}}_p^\star =\mathbf{{1}}_p/\sqrt{p}$ and $\mathbf{{U}} = {\mathbf{{Q}}}^{\scriptscriptstyle \top }\big[\mathbf{{1}}_p^\star \ \varvec{\Lambda }\big]$ is a $(k+1)\times (k+1)$ upper triangular matrix in which $\mathbf{{Q}}=\big[\mathbf{{q}}_1 \ \mathbf{{q}}_2 \, \ldots \, \mathbf{{q}}_{k+1}\big]$ is a $p\times (k+1)$ matrix with orthonormal columns given by:

38 $$\begin{aligned} \mathbf{{q}}_1= \mathbf{{1}}_p^\star \qquad \text {and} \qquad \mathbf{{q}}_{l+1}=\big (\varvec{\lambda }_l - \sum _{h=1}^{l}\mathbf{{q}}_h{\varvec{\lambda }}_l^{\scriptscriptstyle \top }\mathbf{{q}}_{h}\big )/c_{l+1}, \end{aligned}$$

where $l=1,2,\ldots ,k$ and $c_{l+1}$ is a constant chosen to ensure $\mathbf{{q}}_{l+1}$ has unit length.

It is assumed that:

39 $$\begin{aligned} \left[ \begin{array}{c} \varvec{\gamma }_{\mathbf{{g}}} \\ \, \mathbf{{f}}^\star \end{array}\right] \sim \text{ N }\left( \left[ \begin{array}{c} \mathbf{{0}} \\ \mathbf{{0}} \end{array}\right] , \left[ \begin{array}{cc} p\sigma _g^{2} &{} \, \mathbf{{D}}^\star _{12} \\ \mathbf{{D}}^{\star}_{21} &{} \, \mathbf{{D}}^\star _{22} \end{array} \right] \otimes \mathbf{{G_g}}\right) , \end{aligned}$$

where $$\mathbf{{D}}^\star =\mathbf{{U}}\left[ \begin{array}{cc} p\sigma _1^{2} &{} \, \mathbf{{0}} \\ \mathbf{{0}} &{} \, \mathbf{{D}} \end{array} \right] {\mathbf{{U}}}^{\scriptscriptstyle \top }$$,   $\sigma _g^{2}=\sigma _1^{2} + {\sum _{l=1}^kd_l{\bar{\lambda }}^2_l}$ and $\bar{\lambda }_l=\mathbf{{1}}_p^{\scriptscriptstyle \top }\varvec{\lambda }_l/p.$ The FAM*k* variance matrix in Eq. 6 is now given by:

40 $$\mathbf{{G}}= \left( \big[\mathbf{{1}}_p^\star \ \varvec{\Lambda }^{\hskip -1pt \star} \big]\left[ \begin{array}{cc} p\sigma _g^{2} &{} \, \mathbf{{D}}^\star _{12} \\ \mathbf{{D}}^{\star}_{21} &{} \, \mathbf{{D}}^\star _{22} \end{array} \right] \big[\mathbf{{1}}_p^\star \ \varvec{\Lambda }^{\hskip -1pt \star} {\big]}^{{\scriptscriptstyle \top }} + \varvec{\Psi }\right) \otimes \mathbf{{G_g}},$$

where $$\mathbf{G_e} \equiv \sigma_g^2\mathbf{J}_p + \mathbf{1}_p^{\star}{\mathbf{D}}^{\star}_{12}\boldsymbol{\Lambda}^{\star {\!\scriptscriptstyle \hskip 0.75pt \top}} + \boldsymbol{\Lambda}^{\star}{\mathbf{D}}^{\star}_{21}\mathbf{1}_p^{\star {\!\scriptscriptstyle \hskip 0.75pt \top}} +\boldsymbol{\Lambda }^{\star}{\mathbf{D}}^{\star}_{22}\boldsymbol{\Lambda}^{\star {\!\scriptscriptstyle \hskip 0.75pt \top}} +\boldsymbol{\Psi}$$.

The conventional FA*k* model can be viewed as a special FAM*k* model where the intercept variance, $\sigma ^2_1$, is constrained to zero. The variance matrix in Eq. 10 can therefore be written as:

41 $$\begin{aligned} \mathbf{{G}} = \left( \big[\mathbf{{1}}^\star _p \ \varvec{\Lambda }\big]\left[ \begin{array}{cc} 0 &{} \ \mathbf{{0}} \\ \mathbf{{0}} &{} \ \mathbf{{D}} \end{array} \right] \big[\mathbf{{1}}^\star _p \ \varvec{\Lambda }{\big]}^{{\scriptscriptstyle \top }} + \varvec{\Psi }\right) \otimes \mathbf{{G_g}}. \end{aligned}$$

Simple main effects can be obtained from this model using a similar Gram-Schmidt process as above. The FA*k* variance matrix in Eq. 41 is now given by:

42 $$\mathbf{{G}}= \left( \big[\mathbf{{1}}_p^\star \ \varvec{\Lambda}^{\hskip -1pt \star} \big]\left[ \begin{array}{cc} p\bar{\lambda }^{2} &{} \, \mathbf{{D}}^\star _{12} \\ \mathbf{{D}}^{\star}_{21} &{} \, \mathbf{{D}}^\star _{22} \end{array} \right] \big[\mathbf{{1}}_p^\star \ \varvec{\Lambda }^{\hskip -1pt \star} {\big]}^{{\scriptscriptstyle \top }} + \varvec{\Psi }\right) \otimes \mathbf{{G_g}},$$

where $$\mathbf{{G}}_{\mathbf{{e}}} \equiv \bar{\lambda }^{2}\mathbf{{J}} _p + {\mathbf{1}}^{\star}_p{\mathbf{D}}^{\star}_{12}{\boldsymbol{\Lambda}}^{\hskip -1pt \star {\scriptscriptstyle \top }} +
{\boldsymbol{\Lambda}}^{\star}{\mathbf{D}}^{\star}_{21}{\mathbf{1}}^{\hskip -1pt \star {\scriptscriptstyle \top }}_p +\varvec{\Lambda }^{\hskip -1pt \star}\mathbf{{D}}_{22}^\star \varvec{\Lambda }^{\hskip -1pt \star {\scriptscriptstyle \top }} + \varvec{\Psi }$$ and $$\bar{\lambda }^{2} = {\sum _{l=1}^kd_l{\bar{\lambda }}^2_l}$$ is the simple main effect variance, which is equal to $${\sigma }_g^{2}$$ in Eq. 40 when $${\sigma ^2_1}=0.$$

The FA*k* model in Eq. 10 can therefore be written as:

43 $$\begin{aligned} \mathbf{{u}} = \big (\mathbf{{1}}_p^\star \otimes \mathbf{{I}}_v \big )\varvec{\gamma }_{\mathbf{{g}}} + \big (\varvec{\Lambda }^{\hskip -1pt\star} \otimes \mathbf{{I}}_v \big )\mathbf{{f}}^\star + \varvec{\delta }, \end{aligned}$$

where $\varvec{\gamma }_{\mathbf{{g}}}$ is a *v*-vector of simple main effects, with:

44 $$\begin{aligned} \varvec{\gamma }_{\mathbf{{g}}} = \sqrt{p}\,{\sum _{l=1}^k\bar{\lambda }_l}\mathbf{{f}}_l \qquad \text {and} \qquad \varvec{\gamma }_{\mathbf{{g}}} \sim \text{ N }\left( \mathbf{{0}}, \, p\bar{\lambda }^{2} \mathbf{{G_g}}\right) . \end{aligned}$$

### Principal component rotation

Constraints are required in the FAM-LMM and FA-LMM during estimation to ensure unique solutions for ${\varvec{\Lambda }}$ and ${\mathbf{{D}}}$. Following Smith et al. (2021), the upper right elements of ${\varvec{\Lambda }}$ are set to zero when $k>1$ and ${\mathbf{{D}}}$ is set to $\mathbf{{I}}_k$. Let the loadings and scores with these constraints be denoted by ${\varvec{\Lambda }}^{\hskip -1pt*}$ and ${\mathbf{{f}}}^*$, with ${\mathbf{{f}}}^* \sim \text{ N }\big (\mathbf{{0}}, \, \mathbf{{I}}_k \otimes \mathbf{{G_g}}\big )$. The loadings and scores can be rotated back to their original form for interpretation. This rotation is given by:

45 $$\begin{aligned} {\varvec{\Lambda }} = {\varvec{\Lambda }}^{\hskip -1pt *}{\mathbf{{V}}}{\mathbf{{D}}}^{-1/2} \qquad \text {and} \qquad {\mathbf{{f}}} = \big ({\mathbf{{D}}}^{1/2}{\mathbf{{V}}}^{\scriptscriptstyle \top }\otimes \mathbf{{I}}_v\big ){\mathbf{{f}}}^*, \end{aligned}$$

where ${\mathbf{{V}}}$ is a $k\times k$ orthonormal matrix of right singular vectors and ${\mathbf{{D}}}^{1/2}=\oplus _{l=1}^{k}\sqrt{d_l}$ is a diagonal matrix of singular values sorted in decreasing order, with ${\mathbf{{f}}} \sim \text{ N }\big (\mathbf{{0}}, \, {\mathbf{{D}}} \otimes \mathbf{{G_g}}\big )$. These matrices are obtained from the singular value decomposition ${\varvec{\Lambda }}^{\hskip -1pt*} = {\mathbf{{U}}}{\mathbf{{D}}}^{1/2}{\mathbf{{V}}}^{\scriptscriptstyle \top }$, where ${\mathbf{{U}}}$ is a $p\times k$ orthonormal matrix of left singular vectors and ${\varvec{\Lambda }} \equiv {\mathbf{{U}}}$ in Eq. 45.

The loadings and scores can then be rotated using the Gram-Schmidt process in the previous section to obtain simple main effects for either model. Alternatively, generalised main effects can be obtained for the FA-LMM using Eq. 11. In terms of the FAM-LMM, however, an alternative rotation is required which consumes the intercept variance, $\sigma ^2_1$, into the factors. This rotation is given by:

$$\begin{aligned} {\varvec{\Lambda }}^{\hskip -0.75pt \large{\bullet }} = \big [\mathbf{{1}}^\star _p \ {\varvec{\Lambda }}\big ]{\mathbf{{V}}}^{\large{\bullet }}{\mathbf{{D}}}^{{\large\bullet }-1/2} \ \ \ \text {and} \ \ \ {\mathbf{{f}}}^{\large{\bullet }} = \big ({\mathbf{{D}}}^{{\large\bullet }1/2}{\mathbf{{V}}}^{\large{\bullet }{\scriptscriptstyle \top }} \otimes \mathbf{{I}}_v\big )\left( \begin{array}{c} \varvec{\gamma }_{\mathbf{{1}}} \\ \mathbf{{f}} \end{array}\right) , \end{aligned}$$

where ${\mathbf{{V}}}^{\large{\bullet }}$ is a $(k+1)\times (k+1)$ orthonormal matrix and ${\mathbf{{D}}}^{{\large\bullet} 1/2}=\oplus _{l=1}^{k+1}\sqrt{d^{\large{\bullet }}_l}$ is a diagonal matrix, with ${\mathbf{{f}}}^{\large{\bullet }} \sim \text{ N }\big (\mathbf{{0}}, \, {\mathbf{{D}}}^{\large{\bullet }} \otimes \mathbf{{G_g}}\big )$. These matrices are obtained from the singular value decomposition $\big [\mathbf{{1}}^\star _p \ {\varvec{\Lambda }}\big ] = {\mathbf{{U}}}^{\large{\bullet }}{\mathbf{{D}}}^{{\large\bullet }1/2}{\mathbf{{V}}}^{\large{\bullet }{\scriptscriptstyle \top }}$, where ${\mathbf{{U}}}^{\large{\bullet }}$ is a $p\times (k+1)$ orthonormal matrix and ${\varvec{\Lambda }}^{\hskip -0.5pt \large{\bullet }} \equiv {\mathbf{{U}}}^{\large{\bullet }}$.

The FAM*k* model in Eq. 5 can therefore be written as:

46 $$\begin{aligned} \mathbf{{u}} = \big (\varvec{\Lambda }^{\hskip -0.75pt {\large\bullet }} \otimes \mathbf{{I}}_v \big )\mathbf{{f}}^{\large{\bullet }} + \varvec{\delta }, \end{aligned}$$

where $\varvec{\Lambda }^{\hskip -0.75pt \large{\bullet }}$ is a $p\times (k+1)$ matrix and $\mathbf{{f}}^{\large{\bullet }}$ is a $v(k+1)$-vector. The generalised main effects are based on the first factor, with:

47 $$\begin{aligned} \varvec{\gamma }_{\mathbf{{g}}}^{{\star }} = \bar{\lambda }_1^{\large{\bullet }}\mathbf{{f}}^{\large{\bullet }}_1 \ \qquad \text {and} \ \qquad \varvec{\gamma }_{\mathbf{{g}}}^{{\star }} \sim \text{ N }\left( \mathbf{{0}}, \, d_1^{\large{\bullet }}\bar{\lambda }_1^{{\large\bullet }2} \mathbf{{G_g}}\right) , \end{aligned}$$

where $$\bar{\lambda }_1^{\large{\bullet }}=\mathbf{{1}}_p^{\scriptscriptstyle \top }\varvec{\lambda }^{\large{\bullet }}_1/p$$.

## Code availability

The *R* scripts to fit all linear mixed models in Table 3 using *ASReml-R* are provided in the Supplementary Material.
