# Correction to: Genomic selection using random regressions on known and latent environmental covariates

**DOI:** 10.1007/s00122-023-04417-8

**Published:** 2023-08-04

**Authors:** Daniel J. Tolhurst, R. Chris Gaynor, Brian Gardunia, John M. Hickey, Gregor Gorjanc

**Affiliations:** 1 The Roslin Institute and Royal (Dick) School of Veterinary Studies, University of Edinburgh, Easter Bush, UK; 2 Cotton Product Design, Bayer CropScience, St Louis, USA; 3 Corn Product Design, Bayer CropScience, Barcelona, Spain

**Correction to: Theoretical and Applied Genetics (2022) 135:3393–3415** 10.1007/s00122-022-04186-w

The formula displayed immediately after Eqs. 7 and 40 has been incorrectly published in the original publication. The complete correct formula is given below.

$$ {\mathbf{G}}_{{\mathbf{e}}} \equiv \sigma_{g}^{2} {\mathbf{J}}_{p} + {\mathbf{1}}_{p}^{{ \star }} {\mathbf{D}}^{{ \star }} _{12} {{\varvec{\Lambda}}}^{{{ \star \top }}} + {{\varvec{\Lambda}}}^{{ \star }} {\mathbf{D}}^{{ \star }} _{21} {\mathbf{1}}_{p}^{{{ \star \top }}} + {{\varvec{\Lambda}}}^{{ \star }} {\mathbf{D}}^{{ \star }}_{22} {{\varvec{\Lambda}}}^{{{ \star \top }}} + {{\varvec{\Psi}}} $$

The original article has been corrected.

