# Supplementary material for: Genomic selection using random regressions on known and latent environmental covariates
Source: Theor Appl Genet. 2022 Sep 6;135(10):3393–415. doi: 10.1007/s00122-022-04186-w (PMC9519718; doi:10.1007/s00122-022-04186-w)
Supplement: Supplementary file 1 — (PDF 496 KB) [file 122_2022_4186_MOESM1_ESM.pdf]

Theoretical and Applied Genetics

Supplementary material: Genomic selection using random regressions on known and latent environmental covariates

Daniel J. Tolhurst · R. Chris Gaynor · Brian Gardunia · John M. Hickey · Gregor Gorjanc

Received: 01 Oct 2021/ Accepted: 28 Jun 2022

Key message The integration of known and latent environmental covariates within a single-stage genomic selection approach provides breeders with an informative and practical framework to utilise genotype by environment interaction for prediction into observed and unobserved environments.

D.J. Tolhurst ✉
The Roslin Institute and Royal (Dick) School of Veterinary Studies, University of Edinburgh, Easter Bush, Midlothian EH25 9RG, United Kingdom
E-mail: D.J.Tolhurst@sms.ed.ac.uk

1 Test data description

1.1 Experimental design and phenotypic data

Table 9 Summary of the 2018 P2 MET dataset for seed cotton yield. Presented for each environment is the number of trials, genotypes (with one replicate) and plots (total and missing), as well as the mean yield (t/ha) and generalised narrow-sense heritability (h^2). *Black lines* distinguish the △ Southeast, ○ Midsouth and × Texas growing regions.

			Genotypes*		Plots		Yield	
State	Env	Trials	Total	1rep	Total	NAs	Mean	h^2
	18GA1	1	55	55	55	0	1.47	0.96
	18GA2	1	55	55	55	0	1.94	0.97
△ Georgia	18GA3	1	53	53	55	2	1.81	0.53
	18MO1	1	55	55	55	0	2.38	0.26
○ Missouri	18MO2	1	55	55	55	0	2.34	0.56
	18AR1	1	49	49	55	6	1.66	0.88
○ Arkansas	18AR2	1	55	55	55	0	2.47	0.34
	18MS1	1	55	55	55	0	2.26	0.32
	18MS2	1	55	55	55	0	2.19	0.55
	18MS3	1	55	55	55	0	1.36	0.91
	18MS4	1	55	55	55	0	1.58	0.82
○ Mississippi	18MS5	1	53	53	55	2	2.22	0.51
	18LA1	1	55	55	55	0	1.30	0.60
○ Louisiana	18LA2	1	55	55	55	0	1.73	0.30
	18TX1	1	54	54	55	1	2.79	0.51
	18TX2	1	39	39	55	6	1.56	0.00
	18TX3	1	54	54	55	1	2.77	0.52
	18TX4	1	55	55	55	0	1.91	0.27
	18TX5	1	54	54	55	1	1.55	0.26
× Texas	18TX6	1	55	55	55	0	2.99	0.10
**Overall**	**-**	**20**	**55**	**-**	**1,110**	**19**	**2.01**	**0.51**

* Total number after missing plots removed.

1.2 Environmental covariate data

Table 10 Summary of the known environmental covariates in the 2018 P2 MET dataset. Presented for each covariate is the minimum, mean and maximum for the △ Southeast, ○ Midsouth and × Texas growing regions.

		△ Southeast			○ Midsouth			× Texas		
Covariate	Description	Min	Mean	Max	Min	Mean	Max	Min	Mean	Max
LAT	latitude	31.0	31.5	32.2	31.6	34.0	37.0	32.8	33.2	33.6
LONG	longitude	-84.7	-83.8	-83.0	-91.9	-90.8	-89.6	-102.5	-101.6	-99.8
avgCCR	average cloud cover	0.60	0.60	0.60	0.52	0.54	0.56	0.43	0.44	0.47
maxDPT	max dew point temperature	22.0	22.4	22.9	19.7	21.4	22.4	15.7	16.4	18.4
maxDSR	max downward solar radiation	2.73	2.76	2.79	2.69	2.76	2.81	2.92	3.00	3.06
minHUM	min humidity	45.2	47.0	49.7	47.5	51.4	54.5	29.9	33.6	37.6
maxNSR	max net solar radiation	2.28	2.30	2.33	2.21	2.30	2.38	2.37	2.39	2.43
maxPRP	max precipitation	0.05	0.07	0.08	0.03	0.04	0.06	0.03	0.03	0.03
totPRP	total precipitation	3.17	3.31	3.53	2.45	3.05	3.69	1.73	2.00	2.60
maxTMP	max temperature	31.7	32.0	32.4	28.9	30.3	32.1	30.4	31.3	32.2
minTMP	min temperature	21.4	21.6	22.0	19.2	20.9	22.1	18.3	19.0	21.0
minWSP	min wind speed	8.49	8.71	8.90	8.23	8.88	9.74	15.23	15.64	16.23
avgWDR	average wind direction	174.9	178.3	182.1	154.8	161.9	169.5	150.2	153.1	154.1
maxST1	max soil temperature 1	31.0	31.6	32.0	28.6	30.1	31.8	32.3	34.2	36.4
minST1	min soil temperature 1	23.0	23.4	24.1	20.3	22.0	23.4	21.2	22.0	23.6
avgSM3	soil moisture 3	0.05	0.20	0.27	0.18	0.25	0.29	0.09	0.16	0.22
avgSM4	soil moisture 4	0.09	0.25	0.32	0.30	0.34	0.37	0.07	0.13	0.17
minST4	min soil temperature 4	21.5	22.3	23.4	19.6	21.8	23.4	22.9	23.6	25.1

Note: Values presented are prior to centring and scaling.

2 Integrated factor analytic model

2.1 Statistical models

The integrated factor analytic (IFAk) model is an extension of the FARk model to include a generalised set of main effects, instead of simple main effects. The IFAk model can also be viewed as a special FAk model with loadings constrained to be linear combinations of two orthogonal sources of GEI, that is known and latent environmental covariates. The loadings matrix in Equation 10 of the manuscript can therefore be written as:

$$\begin{aligned}\boldsymbol{\Lambda} &= \mathbf{S}\boldsymbol{\Lambda}_\mathbf{s} + \boldsymbol{\Gamma}\boldsymbol{\Lambda}_\mathbf{r} \qquad \text{or} \qquad & \boldsymbol{\Lambda} &= [\mathbf{S}\boldsymbol{\Lambda}_\mathbf{s}\ \ \boldsymbol{\Gamma}\boldsymbol{\Lambda}_\mathbf{r}] \\ &= \mathbf{B}\begin{bmatrix}\boldsymbol{\Lambda}_\mathbf{s}\\ \boldsymbol{\Lambda}_\mathbf{r}\end{bmatrix} & &= \mathbf{B}\begin{bmatrix}\boldsymbol{\Lambda}_\mathbf{s} & \mathbf{0}\\ \mathbf{0} & \boldsymbol{\Lambda}_\mathbf{r}\end{bmatrix},\end{aligned} \tag{48}$$

where $\mathbf{B} = [\mathbf{S}\ \ \boldsymbol{\Gamma}]$ is a $p \times p$ matrix of basis functions (assumed to have full rank), $\mathbf{S} = [\mathbf{s}_1\ \mathbf{s}_2\ \ldots\ \mathbf{s}_q]$ is the $p \times q$ matrix of known environmental covariates and $\boldsymbol{\Gamma} = [\mathbf{r}_1\ \mathbf{r}_2\ \ldots\ \mathbf{r}_{p-q}]$ is a $p\times(p-q)$ orthogonal projection matrix, such that $\mathbf{S}^\top\boldsymbol{\Gamma} = \mathbf{0}$.

The two loadings matrices in Equation 48 correspond to the dependent and independent formulations of the IFAk model. The dependent formulation of the IFAk model was presented in Equation 19. The independent formulation constructs a separate regression across the known and latent environmental covariates, with separate factor loadings given by:

$$\boldsymbol{\Lambda}_\mathbf{s} = \left[\boldsymbol{\lambda}_{\mathbf{s}_1}\ \boldsymbol{\lambda}_{\mathbf{s}_2}\ \ldots\ \boldsymbol{\lambda}_{\mathbf{s}_{k_s}}\right] \quad \text{and} \quad \boldsymbol{\Lambda}_\mathbf{r} = \left[\boldsymbol{\lambda}_{\mathbf{r}_1}\ \boldsymbol{\lambda}_{\mathbf{r}_2}\ \ldots\ \boldsymbol{\lambda}_{\mathbf{r}_{k_r}}\right],$$

where $\boldsymbol{\Lambda}_\mathbf{s}$ is a $q\times k_s$ matrix corresponding to the known covariates and $\boldsymbol{\Lambda}_\mathbf{r}$ is a $(p-q)\times k_r$ matrix corresponding to the latent covariates. The common factors underlying $\boldsymbol{\Lambda}_\mathbf{s}$ and $\boldsymbol{\Lambda}_\mathbf{r}$ are therefore referred to as the *separate* set of known and latent factors, respectively.

The independent formulation is obtained by substituting the second loadings matrix in Equation 48 into Equation 10, which gives:

$$\mathbf{u} = \left(\mathbf{S}\boldsymbol{\Lambda}_\mathbf{s} \otimes \mathbf{I}_v\right)\mathbf{f}_\mathbf{s} + \left(\boldsymbol{\Gamma}\boldsymbol{\Lambda}_\mathbf{r} \otimes \mathbf{I}_v\right)\mathbf{f}_\mathbf{r} + \boldsymbol{\delta}. \tag{49}$$

where $\mathbf{f}_\mathbf{s} = \left(\mathbf{f}_{\mathbf{s}_1}^\top, \mathbf{f}_{\mathbf{s}_2}^\top, \ldots, \mathbf{f}_{\mathbf{s}_{k_s}}^\top\right)^\top$ is a vk_s-vector corresponding to the known covariates and $\mathbf{f}_\mathbf{r} = \left(\mathbf{f}_{\mathbf{r}_1}^\top, \mathbf{f}_{\mathbf{r}_2}^\top, \ldots, \mathbf{f}_{\mathbf{r}_{k_r}}^\top\right)^\top$ is a vk_r-vector corresponding to the latent covariates. The model in Equation 49 is referred to as the IFAk_s-k_r model.

It is assumed that:

$$\begin{bmatrix}\mathbf{f}_\mathbf{s}\\ \mathbf{f}_\mathbf{r}\end{bmatrix} \sim \mathrm{N}\left(\begin{bmatrix}\mathbf{0}\\ \mathbf{0}\end{bmatrix}, \begin{bmatrix}\mathbf{D}_\mathbf{s} & \mathbf{0}\\ \mathbf{0} & \mathbf{D}_\mathbf{r}\end{bmatrix} \otimes \mathbf{G}_\mathbf{g}\right),$$

where $\mathbf{D}_\mathbf{s} = \oplus_{l=1}^{k_s} d_{s_l}$ and $\mathbf{D}_\mathbf{r} = \oplus_{l=1}^{k_r} d_{r_l}$ are the score variance matrices with diagonal elements ordered as $d_{s_1} > d_{s_2} > \ldots > d_{s_{k_s}}$ and $d_{r_1} > d_{r_2} > \ldots > d_{r_{k_r}}$, respectively.

The variance matrix for $\mathbf{u}$ is therefore given by:

$$\mathbf{G} = \left(\mathbf{B}\begin{bmatrix}\boldsymbol{\Lambda}_\mathbf{s}\mathbf{D}_\mathbf{s}\boldsymbol{\Lambda}_\mathbf{s}^\top & \mathbf{0}\\ \mathbf{0} & \boldsymbol{\Lambda}_\mathbf{r}\mathbf{D}_\mathbf{r}\boldsymbol{\Lambda}_\mathbf{r}^\top\end{bmatrix}\mathbf{B}^\top + \boldsymbol{\Psi}\right) \otimes \mathbf{G}_\mathbf{g}, \tag{50}$$

where $\mathbf{G}_\mathbf{e} \equiv (\mathbf{S}\boldsymbol{\Lambda}_\mathbf{s}\mathbf{D}_\mathbf{s}\boldsymbol{\Lambda}_\mathbf{s}^\top\mathbf{S}^\top + \boldsymbol{\Gamma}\boldsymbol{\Lambda}_\mathbf{r}\mathbf{D}_\mathbf{r}\boldsymbol{\Lambda}_\mathbf{r}^\top\boldsymbol{\Gamma}^\top + \boldsymbol{\Psi})$. This variance matrix is not equivalent to the conventional FAk model, however, it does have the same number of variance parameters when $k_s = k_r$.

Alternatively, the projection matrix can be removed from Equation 49, which gives:

$$\mathbf{u} = \left(\mathbf{S}\boldsymbol{\Lambda}_\mathbf{s} \otimes \mathbf{I}_v\right)\mathbf{f}_\mathbf{s} + \left(\boldsymbol{\Lambda}_\mathbf{r} \otimes \mathbf{I}_v\right)\mathbf{f}_\mathbf{r} + \boldsymbol{\delta}, \tag{51}$$

such that $\boldsymbol{\Lambda}_\mathbf{r}$ is now a $p \times k$ matrix. Note that this formulation has more variance parameters than the conventional FAk model.

2.2 Model fitting

Table 11 Independent formulation of the IFA-LMM, **a.** with or **b.** without the projection matrix. Presented for each model is the number of estimated genetic variance parameters, residual log-likelihood, AIC and percentage of variance explained by the known covariates ($\bar{v}_s$) and overall ($\bar{v}$).

Independent formulation of the IFA-LMM											
**a.** With projection matrix						**b.** Without projection matrix					
Model	Pars	Loglik	AIC	$\bar{v}_s$	$\bar{v}$	Model	Pars	Loglik	AIC	$\bar{v}_s$	$\bar{v}$
diag	24	10,249.3	-20,194.7	-	-	*diag*	24	10,249.3	-20,194.7	-	-
IFA1-1	48	10,767.8	-21,183.6	16.7	50.4	IFA1-1	48	10,667.1	-20,982.2	7.0	43.2
IFA2-2	70	10,879.7	-21,363.5	28.8	68.2	IFA2-2	71	10,827.4	-21,256.8	20.1	60.4
IFA3-2	86	10,907.7	-21,387.4	33.1	72.5	IFA3-3	93	10,940.3	-21,438.5	30.1	70.7
**IFA4-2**	**101**	**10,931.3**	**-21,404.6**	**36.4**	**76.9**	**IFA4-3**	**108**	**10,971.9**	**-21,471.9**	**34.4**	**74.9**
IFA5-2	115	10,954.1	-21,422.3	40.0	81.3	IFA5-3	122	10,996.4	-21,492.8	36.2	78.0

Note: 128 non-genetic and residual variance parameters estimated in all models. The selected independent formulation of the IFA4-2 model with projection matrix and IFA3-2 model without projection matrix are distinguished with *bold font.*

2.3 Model assessment

Table 12 Summary of the prediction accuracy for the 2017 current and 2018 future environments. Presented for each model is the minimum, mean and maximum prediction accuracy for the △ Southeast, ○ Midsouth and × Texas, as well as overall across all growing regions.

		△ Southeast			○ Midsouth			× Texas			Overall		
Year	Model	Min	Mean	Max	Min	Mean	Max	Min	Mean	Max	Min	Mean	Max
	$rreg_1$	0.27	0.51	0.68	0.30	0.58	0.77	0.27	0.47	0.60	0.27	0.52	0.77
	$rreg_2$	0.27	0.52	0.69	0.29	0.58	0.76	0.27	0.47	0.61	0.27	0.52	0.76
	FAR4	0.25	0.50	0.66	0.34	0.59	0.77	0.25	0.48	0.64	0.25	0.52	0.77
	IFA4-2_1	0.33	**0.61**	**0.77**	0.43	0.67	0.80	0.28	0.49	0.64	0.28	0.59	0.80
	IFA3-2_2	**0.37**	0.60	0.75	**0.49**	**0.69**	**0.81**	0.28	**0.50**	**0.65**	0.28	**0.60**	**0.81**
2017	IFA4-3	0.33	0.60	0.76	0.45	0.68	0.79	**0.29**	**0.50**	**0.65**	**0.29**	**0.60**	0.79
	$rreg_1$	0.58	0.60	0.64	0.30	0.50	0.71	-0.03	0.20	0.34	-0.03	0.42	0.71
	$rreg_2$	0.58	0.61	0.64	0.28	0.49	0.70	-0.02	0.21	0.36	-0.02	0.42	0.70
	FAR4	0.58	0.61	0.67	0.26	0.49	0.71	0.02	0.22	0.36	0.02	0.43	0.71
	IFA4-2_1	0.59	0.64	0.66	0.20	0.55	0.75	0.18	0.40	0.57	0.18	0.52	0.75
	IFA3-2_2	**0.62**	0.66	0.68	0.23	0.56	0.75	**0.30**	0.37	**0.62**	0.23	0.52	0.75
2018	IFA4-3	0.60	**0.67**	**0.71**	**0.31**	**0.60**	**0.79**	**0.30**	**0.44**	**0.62**	**0.30**	**0.56**	**0.79**

Note: $rreg_1$ and $rreg_2$ are the random regression models in Jarquín et al. (2014) and Heslot et al. (2014). The IFA4-3 model is the dependent formulation in Equation 22 of the manuscript. The IFA4-2_1 model is the independent formulation in Equation 49 and the IFA3-2_2 model is the independent formulation without projection matrix in Equation 51. The highest accuracy is distinguished with *bold font*.

3 Sparse implementation of the IFA-LMM

This section extends the sparse implementation of the average information (AI) algorithm for the integrated factor analytic linear mixed model (IFA-LMM). Thompson et al. (2003) introduced the sparse implementation for factor analytic linear mixed models as a more efficient approach to estimate the key variance parameters, that is the loadings, score variances and specific variances. This approach also provides a natural way to handle cases where some of the specific variances are zero. When all specific variances are zero, this leads to a (fully) reduced rank factor analytic model (Kirkpatrick and Meyer, 2004).

3.1 Preliminaries

The IFA-LMM for $\mathbf{y}$, the n-vector of phenotypic data on v genotypes and p environments with q known covariates, is given by:

$$\mathbf{y} = \mathbf{X}\boldsymbol{\tau} + \mathbf{Z}_{\boldsymbol{\Lambda}_\mathbf{b}}\mathbf{f} + \mathbf{Z}\boldsymbol{\delta} + \boldsymbol{\varepsilon}, \tag{52}$$

where $\mathbf{Z}_{\boldsymbol{\Lambda}_\mathbf{b}} = \mathbf{Z}\left(\mathbf{B}\boldsymbol{\Lambda}_\mathbf{b} \otimes \mathbf{I}_v\right)$ is the $n \times vk$ integrated factor analytic design matrix, $\mathbf{B} = [\mathbf{S}\ \boldsymbol{\Gamma}]$ is the $p \times p$ matrix of basis functions, $\boldsymbol{\Lambda}_\mathbf{b} = \begin{bmatrix}\boldsymbol{\Lambda}_\mathbf{s}\\ \boldsymbol{\Lambda}_\mathbf{r}\end{bmatrix}$ is the $p \times k$ matrix of joint factor loadings and $\boldsymbol{\varepsilon} = \mathbf{Z}_\mathbf{p}\mathbf{u}_\mathbf{p} + \mathbf{e}$, with $\mathrm{var}(\boldsymbol{\varepsilon}) = \mathbf{R}_{\boldsymbol{\varepsilon}}$. The IFA-LMM in Equation 52 can be extended by partitioning the regression residuals as $\boldsymbol{\delta} = (\boldsymbol{\delta}_1^\top, \boldsymbol{\delta}_2^\top)^\top$, where $\boldsymbol{\delta}_1$ is a vp_1-vector with no zero elements and $\boldsymbol{\delta}_2$ is a vp_2-vector with all zero elements, such that $p = p_1 + p_2$. Two simpler models can also be obtained:

1. When $p_1 = p$ and $p_2 = 0$, no specific variances are zero. This model is the conventional IFA-LMM.
2. When $p_1 = 0$ and $p_2 = p$, all specific variances are zero. This model is referred to as the reduced rank IFA-LMM.

The model considered below allows some specific variances to be non-zero and some to be zero. The IFA-LMM can therefore be written as:

$$\mathbf{y} = \mathbf{X}\boldsymbol{\tau} + \mathbf{Z}_{\boldsymbol{\Lambda}_\mathbf{b}}\mathbf{f} + \mathbf{Z}_1\boldsymbol{\delta}_1 + \mathbf{Z}_2\boldsymbol{\delta}_2 + \boldsymbol{\varepsilon}, \tag{53}$$

where $\mathbf{Z} = [\mathbf{Z}_1\ \mathbf{Z}_2]$ is partitioned conformably with $\boldsymbol{\delta}$.

It is assumed that $\boldsymbol{\delta}_2 = \mathbf{0}$, and that:

$$\begin{bmatrix}\mathbf{f}\\ \boldsymbol{\delta}_1\end{bmatrix} \sim \mathrm{N}\left(\begin{bmatrix}\mathbf{0}\\ \mathbf{0}\end{bmatrix}, \begin{bmatrix}\mathbf{D} & \mathbf{0}\\ \mathbf{0} & \boldsymbol{\Psi}_1\end{bmatrix} \otimes \mathbf{G}_\mathbf{g}\right).$$

In this model, the genotype scores and regression residuals are assumed to be independent. Thompson et al. (2003) also present an equivalent formulation where the random effects are assumed to be dependent, but this formulation is not considered further.

The mixed model equations for the IFA-LMM in Equation 52 are given by:

$$\begin{bmatrix}\mathbf{X}^\top\mathbf{R}_{\boldsymbol{\varepsilon}}^{-1}\mathbf{X} & \mathbf{X}^\top\mathbf{R}_{\boldsymbol{\varepsilon}}^{-1}\mathbf{Z}_{\boldsymbol{\Lambda}_\mathbf{b}} & \mathbf{X}^\top\mathbf{R}_{\boldsymbol{\varepsilon}}^{-1}\mathbf{Z}_1\\ \mathbf{Z}_{\boldsymbol{\Lambda}_\mathbf{b}}^\top\mathbf{R}_{\boldsymbol{\varepsilon}}^{-1}\mathbf{X} & \mathbf{Z}_{\boldsymbol{\Lambda}_\mathbf{b}}^\top\mathbf{R}_{\boldsymbol{\varepsilon}}^{-1}\mathbf{Z}_{\boldsymbol{\Lambda}_\mathbf{b}} + \mathbf{D}^{-1}\otimes\mathbf{G}_\mathbf{g}^{-1} & \mathbf{Z}_{\boldsymbol{\Lambda}_\mathbf{b}}^\top\mathbf{R}_{\boldsymbol{\varepsilon}}^{-1}\mathbf{Z}_1\\ \mathbf{Z}_1^\top\mathbf{R}_{\boldsymbol{\varepsilon}}^{-1}\mathbf{X} & \mathbf{Z}_1^\top\mathbf{R}_{\boldsymbol{\varepsilon}}^{-1}\mathbf{Z}_{\boldsymbol{\Lambda}} & \mathbf{Z}_1^\top\mathbf{R}_{\boldsymbol{\varepsilon}}^{-1}\mathbf{Z}_1 + \boldsymbol{\Psi}_1^{-1}\otimes\mathbf{G}_\mathbf{g}^{-1}\end{bmatrix}\begin{bmatrix}\hat{\boldsymbol{\tau}}\\ \tilde{\mathbf{f}}\\ \tilde{\boldsymbol{\delta}}_1\end{bmatrix} = \begin{bmatrix}\mathbf{X}^\top\mathbf{R}_{\boldsymbol{\varepsilon}}^{-1}\mathbf{y}\\ \mathbf{Z}_{\boldsymbol{\Lambda}_\mathbf{b}}^\top\mathbf{R}_{\boldsymbol{\varepsilon}}^{-1}\mathbf{y}\\ \mathbf{Z}_1^\top\mathbf{R}_{\boldsymbol{\varepsilon}}^{-1}\mathbf{y}\end{bmatrix}. \tag{54}$$

3.2 Prediction of genotype scores and regression residuals

Following Smith et al. (2019), Equations 53 and 54 can be written as:

$$\mathbf{y} = \mathbf{W}\boldsymbol{\beta} + \boldsymbol{\varepsilon} \quad \text{and} \quad \mathbf{C}\tilde{\boldsymbol{\beta}} = \mathbf{W}^\top\mathbf{R}_{\boldsymbol{\varepsilon}}^{-1}\mathbf{y}, \tag{55}$$

where $\mathbf{W} = [\mathbf{X}\ \mathbf{Z}_{\boldsymbol{\Lambda}_\mathbf{b}}\ \mathbf{Z}_1]$ and $\mathbf{C} = \mathbf{W}^\top\mathbf{R}_{\boldsymbol{\varepsilon}}^{-1}\mathbf{W} + \mathbf{G}_\mathbf{c}^{-1}$, with:

$$\tilde{\boldsymbol{\beta}} = \begin{pmatrix}\hat{\boldsymbol{\tau}}\\ \tilde{\mathbf{f}}\\ \tilde{\boldsymbol{\delta}}_1\end{pmatrix} \text{ and } \mathbf{G}_\mathbf{c}^{-1} = \begin{bmatrix}\mathbf{0} & \mathbf{0} & \mathbf{0}\\ \mathbf{0} & \mathbf{D}^{-1}\otimes\mathbf{G}_\mathbf{g}^{-1} & \mathbf{0}\\ \mathbf{0} & \mathbf{0} & \boldsymbol{\Psi}_1^{-1}\otimes\mathbf{G}_\mathbf{g}^{-1}\end{bmatrix}. \tag{56}$$

The BLUPs of the key random effects are obtained via absorption of $\mathbf{C}$ onto $\mathbf{y}^\top\mathbf{R}_{\boldsymbol{\varepsilon}}^{-1}\mathbf{y}$, which gives:

$$\tilde{\mathbf{f}} = \left[\mathbf{D}\otimes\mathbf{G}_\mathbf{g}\right]\mathbf{Z}_{\boldsymbol{\Lambda}_\mathbf{b}}^\top\mathbf{P}\mathbf{y} \quad \text{and} \quad \tilde{\boldsymbol{\delta}}_1 = \left[\boldsymbol{\Psi}_1\otimes\mathbf{G}_\mathbf{g}\right]\mathbf{Z}_1^\top\mathbf{P}\mathbf{y}, \tag{57}$$

where $\mathbf{P} = \mathbf{R}_{\boldsymbol{\varepsilon}}^{-1} - \mathbf{R}_{\boldsymbol{\varepsilon}}^{-1}\mathbf{W}\mathbf{C}^{-1}\mathbf{W}^\top\mathbf{R}_{\boldsymbol{\varepsilon}}^{-1}$ is the $n \times n$ residual sum of squares matrix. Absorption of $\mathbf{C}$ also produces the prediction error variance matrices, with:

$$\mathrm{var}(\tilde{\mathbf{f}} - \mathbf{f}) = \mathbf{C}^{\tilde{\mathbf{f}}\tilde{\mathbf{f}}} \quad \text{and} \quad \mathrm{var}(\tilde{\boldsymbol{\delta}}_1 - \boldsymbol{\delta}_1) = \mathbf{C}^{\tilde{\boldsymbol{\delta}}_1\tilde{\boldsymbol{\delta}}_1}, \tag{58}$$

where $\mathbf{C}^{\tilde{\mathbf{f}}\tilde{\mathbf{f}}} = \left[\mathbf{D}\otimes\mathbf{G}_\mathbf{g}\right]\left(\mathbf{I}_{vk}\mathbf{Z}_{\boldsymbol{\Lambda}_\mathbf{b}}^\top\mathbf{P}\mathbf{Z}_{\boldsymbol{\Lambda}_\mathbf{b}}\left[\mathbf{D}\otimes\mathbf{G}_\mathbf{g}\right]\right)$ and $\mathbf{C}^{\tilde{\boldsymbol{\delta}}_1\tilde{\boldsymbol{\delta}}_1} = \left[\boldsymbol{\Psi}_1\otimes\mathbf{G}_\mathbf{g}\right]\left(\mathbf{I}_{vp} - \mathbf{Z}_1^\top\mathbf{P}\mathbf{Z}_1\left[\boldsymbol{\Psi}_1\otimes\mathbf{G}_\mathbf{g}\right]\right)$. These matrices are equivalent to the diagonal blocks in $\mathbf{C}^{-1}$ corresponding to $\tilde{\mathbf{f}}$ and $\tilde{\boldsymbol{\delta}}_1$, respectively.

Lastly, note that the components in Equations 57 and 58 assume that the variance parameters are known. The variance parameters are unknown, however, so they must be estimated. The resulting predictions are therefore referred to as empirical BLUPs.

3.3 Estimation of loadings and specific variances

The REML estimates of the key variance parameters are obtained by maximising the residual log-likelihood, which is given by:

$$\begin{aligned} l(\mathbf{y_2}) &= -\tfrac{1}{2}\left(\log|\mathbf{H}| + \log|\mathbf{X}^\top\mathbf{H}^{-1}\mathbf{X}| + \mathbf{y}^\top\mathbf{P}\mathbf{y}\right)\\ &= -\tfrac{1}{2}\left(\log|\mathbf{H}| + \log|\mathbf{X}^\top\mathbf{H}^{-1}\mathbf{X}| + \mathbf{y}_\mathbf{r}^\top\mathbf{H}^{-1}\mathbf{y}_\mathbf{r}\right), \end{aligned} \tag{59}$$

where $\mathbf{y_2} = \mathbf{L}_2^\top\mathbf{y}$ such that $\mathbf{L}_2^\top\mathbf{X} = \mathbf{0}$ and $\mathbf{y}_\mathbf{r} = \mathbf{y} - \mathbf{X}\hat{\boldsymbol{\tau}}$, with $\mathrm{var}(\mathbf{y}_\mathbf{r}) = \mathbf{H}$ (Verbyla, 1990). In particular, the REML estimates are obtained by solving a set of (score) equations, which are given by:

$$s(\boldsymbol{\kappa}) = \frac{\partial l(\mathbf{y_2})}{\partial \boldsymbol{\kappa}^\top} = \mathbf{0}, \tag{60}$$

where $\boldsymbol{\kappa}$ is the b-vector of variance parameters in the IFA-LMM. The score equations were traditionally solved using numerical approaches based on the observed or expected information. The AI algorithm is based on

the average information, and computes updates as:

$$\boldsymbol{\kappa}^{(m+1)} = \boldsymbol{\kappa}^{(m)} + \left[\boldsymbol{\mathcal{I}}_{\mathbf{a}}^{(m)}\right]^{-1} \mathbf{s}(\boldsymbol{\kappa}^{(m)}), \tag{61}$$

where $\boldsymbol{\kappa}^{(m)}$ is the b-vector of variance parameters, $\boldsymbol{\mathcal{I}}_{\mathbf{a}}^{(m)}$ is the AI matrix and $\mathbf{s}(\boldsymbol{\kappa}^{(m)})$ is the score equations for the mth iteration. Note that the superscript "m" is removed in the following for brevity. The score equation for κ_i is given by (Gilmour et al., 1995):

$$s(\kappa_i) = -\tfrac{1}{2}\left[\mathrm{tr}\big(\mathbf{P}\dot{\mathbf{H}}_i\big) - \mathbf{y}^\top \mathbf{P}\mathbf{q}_i\right], \tag{62}$$

where $\dot{\mathbf{H}}_i = \frac{\partial \mathbf{H}}{\partial \kappa_i}$ and $\mathbf{q}_i$ is the working variate for the ith variance parameter, which is given by:

$$\mathbf{q}_i = \dot{\mathbf{H}}_i \mathbf{P}\mathbf{y}. \tag{63}$$

Next, let the vectors of key variance parameters in the IFA-LMM be denoted by:

$$\boldsymbol{\lambda}_{\mathbf{b}} = \mathrm{vect}(\boldsymbol{\Lambda}_{\mathbf{b}}),\ \mathbf{d} = \mathrm{diag}(\mathbf{D}) \text{ and } \boldsymbol{\psi}_1 = \mathrm{diag}(\boldsymbol{\Psi}_1), \tag{64}$$

where $\boldsymbol{\Lambda}_{\mathbf{b}} = \begin{bmatrix}\boldsymbol{\Lambda}_{\mathbf{s}}\\ \boldsymbol{\Lambda}_{\mathbf{r}}\end{bmatrix}$. The working variates for the key variance parameters can therefore be written as:

$$\mathbf{q}_i = \begin{cases} \mathbf{Z}\left[\mathbf{B}\big(\boldsymbol{\Lambda}_{\mathbf{b}}\mathbf{D}\dot{\boldsymbol{\Lambda}}_{\mathbf{b}_i}^\top + \dot{\boldsymbol{\Lambda}}_{\mathbf{b}_i}\mathbf{D}\boldsymbol{\Lambda}_{\mathbf{b}}^\top\big)\mathbf{B}^\top \otimes \mathbf{G}_{\mathbf{g}}\right]\mathbf{Z}^\top\mathbf{P}\mathbf{y} & \kappa_i \in \boldsymbol{\lambda}_{\mathbf{b}} \\ \mathbf{Z}\left[\mathbf{B}\boldsymbol{\Lambda}_{\mathbf{b}}\dot{\mathbf{D}}_i\boldsymbol{\Lambda}_{\mathbf{b}}^\top\mathbf{B}^\top \otimes \mathbf{G}_{\mathbf{g}}\right]\mathbf{Z}^\top\mathbf{P}\mathbf{y} & \kappa_i \in \mathbf{d} \\ \mathbf{Z}_1\left[\dot{\boldsymbol{\Psi}}_{1_i} \otimes \mathbf{G}_{\mathbf{g}}\right]\mathbf{Z}_1^\top\mathbf{P}\mathbf{y}, & \kappa_i \in \boldsymbol{\psi}_1 \end{cases}$$

where $\dot{\boldsymbol{\Lambda}}_{\mathbf{b}_i} = \frac{\partial \boldsymbol{\Lambda}_{\mathbf{b}}}{\partial \lambda_{b_i}}$, $\dot{\mathbf{D}}_i = \frac{\partial \mathbf{D}}{\partial d_i}$ and $\dot{\boldsymbol{\Psi}}_{1_i} = \frac{\partial \boldsymbol{\Psi}_1}{\partial \psi_{1_i}}$. The working variates for the specific variances can be simplified since the regression residuals are independent across environments. It therefore follows that:

$$\mathbf{q}_i = \mathbf{Z}_{1\cdot i}\mathbf{G}_{\mathbf{g}}\mathbf{Z}_{1_i}^\top\mathbf{P}_{i\cdot}\mathbf{y}, \qquad \kappa_i \in \boldsymbol{\psi}_1$$

where $\mathbf{Z}_{1_i}$ is the $n_i \times v$ design matrix for the ith environment with non-zero specific variance, $\mathbf{P}_{i\cdot}$ are the corresponding n_i rows in $\mathbf{P}$ and $\mathbf{Z}_{1\cdot i}$ are the corresponding v columns in $\mathbf{Z}_1$.

The other important components in Equation 62 are the trace terms. The trace terms for the key variance parameters can be written as:

$$\mathrm{tr}(\mathbf{P}\dot{\mathbf{H}}_i) = \begin{cases} 2\ \mathrm{tr}\left[\big(\dot{\boldsymbol{\Lambda}}_{\mathbf{b}_i}^\top\mathbf{B}^\top \otimes \mathbf{I}_v\big)\mathbf{Z}^\top\mathbf{R}_{\boldsymbol{\varepsilon}}^{-1}\mathbf{W}\mathbf{C}^{\cdot\tilde{\mathbf{f}}}\right] & \kappa_i \in \boldsymbol{\lambda}_{\mathbf{b}} \\ \mathrm{tr}\left[\big(\dot{\mathbf{D}}_i\mathbf{D}^{-1}\boldsymbol{\Lambda}_{\mathbf{b}}^\top\mathbf{B}^\top \otimes \mathbf{I}_v\big)\mathbf{Z}^\top\mathbf{R}_{\boldsymbol{\varepsilon}}^{-1}\mathbf{W}\mathbf{C}^{\cdot\tilde{\mathbf{f}}}\right] & \kappa_i \in \mathbf{d} \\ \mathrm{tr}\left[\big(\dot{\boldsymbol{\Psi}}_{1_i}\boldsymbol{\Psi}_1^{-1} \otimes \mathbf{I}_v\big)\mathbf{Z}_1^\top\mathbf{R}_{\boldsymbol{\varepsilon}}^{-1}\mathbf{W}\mathbf{C}^{\cdot\tilde{\boldsymbol{\delta}}_1}\right], & \kappa_i \in \boldsymbol{\psi}_1 \end{cases}$$

where $\mathbf{C}^{\cdot\tilde{\mathbf{f}}}$ are the vk columns in $\mathbf{C}^{-1}$ corresponding to $\tilde{\mathbf{f}}$ and $\mathbf{C}^{\cdot\tilde{\boldsymbol{\delta}}_1}$ are the vp columns corresponding to $\tilde{\boldsymbol{\delta}}_1$. The trace terms for the specific variances can be further simplified as:

$$\mathrm{tr}(\mathbf{P}\dot{\mathbf{H}}_i) = \tfrac{1}{\psi_{1_i}}\ \mathrm{tr}\left[\mathbf{Z}_{1_i}^\top\mathbf{R}_{\boldsymbol{\varepsilon}_i}^{-1}\mathbf{W}_{i\cdot}\mathbf{C}^{\cdot\tilde{\boldsymbol{\delta}}_{1_i}}\right], \qquad \kappa_i \in \boldsymbol{\psi}_1$$

where $\mathbf{R}_{\boldsymbol{\varepsilon}_i}$ is the ith diagonal block in $\mathbf{R}_{\boldsymbol{\varepsilon}}$, $\mathbf{W}_{i\cdot}$ are the n_i rows in $\mathbf{W}$ corresponding to the ith environment and $\mathbf{C}^{\cdot\tilde{\boldsymbol{\delta}}_{1_i}}$ are the v columns in $\mathbf{C}$ corresponding to $\tilde{\boldsymbol{\delta}}_{1_i}$. The trace terms avoid working with the dense genomic relationship matrix. When $\mathbf{G}_{\mathbf{g}}$ is not prohibitively large, the trace term for the specific variances can also be computed as:

$$\mathrm{tr}(\mathbf{P}\dot{\mathbf{H}}_i) = \tfrac{v}{\psi_{1_i}} - \mathrm{tr}\big[\mathbf{G}_{\mathbf{g}}\mathbf{C}^{\boldsymbol{\eta}_{1_i}\boldsymbol{\eta}_{1_i}}\big], \qquad \kappa_i \in \boldsymbol{\psi}_1$$

where $\mathbf{C}^{\boldsymbol{\eta}_{1_i}\boldsymbol{\eta}_{1_i}}$ is the prediction error variance matrix of $\boldsymbol{\eta}_{1_i} = \mathbf{Z}_{1_i}^\top\mathbf{P}_{i\cdot}\mathbf{y}$.

Lastly, the AI matrix in Equation 61 is given by:

$$\boldsymbol{\mathcal{I}}_{\mathbf{a}} = \tfrac{1}{2}\mathbf{Q}^\top\mathbf{P}\mathbf{Q}, \tag{65}$$

where $\mathbf{Q} = [\mathbf{q}_1\ \mathbf{q}_2\ \ldots\ \mathbf{q}_b]$ is the $n \times b$ matrix of working variates across all b variance parameters. The AI matrix is obtained via absorption of $\mathbf{C}$ onto $\mathbf{Q}^\top\mathbf{R}_{\boldsymbol{\varepsilon}}^{-1}\mathbf{Q}$ (Smith, 1999).

3.4 Trimming the regression residuals

The working variates and trace terms for the specific variances can be further simplified for incomplete data, that is by only considering the regression residuals which correspond to phenotypic data. Mazur (2021) refer to this as trimming.

Let the v-vector of regression residuals for the ith environment with non-zero specific variance be partitioned as $\boldsymbol{\delta}_{1_i} = (\boldsymbol{\delta}_{1_{i;1}}^\top, \boldsymbol{\delta}_{1_{i;0}}^\top)^\top$, where $\boldsymbol{\delta}_{1_{i;1}}$ is the v_i-vector corresponding to genotypes with phenotypic data and $\boldsymbol{\delta}_{1_{i;0}}$ is the $(v - v_i)$-vector corresponding to genotypes without phenotypic data. Also let the $n_i \times v$ design matrix be partitioned conformably as $\mathbf{Z}_{1_i} = [\mathbf{Z}_{1_{i;1}}\ \mathbf{0}]$, where $\mathbf{Z}_{1_{i;1}}$ is a $n_i \times v_i$ matrix.

It is assumed that:

$$\begin{bmatrix}\boldsymbol{\delta}_{1_{i;1}}\\ \boldsymbol{\delta}_{1_{i;0}}\end{bmatrix} \sim \mathrm{N}\left(\begin{bmatrix}\mathbf{0}\\ \mathbf{0}\end{bmatrix}, \psi_{1_i}\begin{bmatrix}\mathbf{G}_{\mathbf{g}_{i;11}} & \mathbf{G}_{\mathbf{g}_{i;10}}\\ \mathbf{G}_{\mathbf{g}_{i;01}} & \mathbf{G}_{\mathbf{g}_{i;00}}\end{bmatrix}\right),$$

Trimming is possible for the regression residuals since $\boldsymbol{\delta}_{1_{i;0}}$ does not contribute to $\mathbf{H}$, and thence does not contribute to the residual log-likelihood. It is therefore more efficient to fit $\mathbf{Z}_{1_{i;1}}\boldsymbol{\delta}_{1_{i;1}}$ for the ith environment instead of $\mathbf{Z}_{1_i}\boldsymbol{\delta}_{1_i}$. The BLUPs of the trimmed regression residuals are then given by:

$$\tilde{\boldsymbol{\delta}}_{1_{i;1}} = \psi_{1_i}\mathbf{G}_{\mathbf{g}_{i;11}}\mathbf{Z}_{1_{i;1}}^\top\mathbf{P}_{i\cdot}\mathbf{y}. \tag{66}$$

The working variates and trace terms in Equation 62 can be written as:

$$\begin{aligned} \mathbf{q}_i &= \mathbf{Z}_{1\cdot i;1}\mathbf{G}_{\mathbf{g}_{i;11}}\mathbf{Z}_{1_{i;1}}^\top\mathbf{P}_{i\cdot}\mathbf{y} \\ \mathrm{tr}(\mathbf{P}\dot{\mathbf{H}}_i) &= \tfrac{1}{\psi_{1_i}}\ \mathrm{tr}\left[\mathbf{Z}_{1_{i;1}}^\top\mathbf{R}_{\boldsymbol{\varepsilon}_i}^{-1}\mathbf{W}_{i\cdot}\mathbf{C}^{\cdot\tilde{\boldsymbol{\delta}}_{1_{i;1}}}\right] \\ &= \tfrac{v_i}{\psi_{1_i}} - \mathrm{tr}\big[\mathbf{G}_{\mathbf{g}_{i;11}}\mathbf{C}^{\boldsymbol{\eta}_{1_{i;1}}\boldsymbol{\eta}_{1_{i;1}}}\big], \qquad \kappa_i \in \boldsymbol{\psi}_1 \end{aligned}$$

where $\mathbf{Z}_{1\cdot i;1}$ are the v_i columns in $\mathbf{Z}_1$ corresponding to $\tilde{\boldsymbol{\delta}}_{1_{i;1}}$, $\mathbf{C}^{\cdot\tilde{\boldsymbol{\delta}}_{1_{i;1}}}$ are the v_i columns in $\mathbf{C}^{\cdot\tilde{\boldsymbol{\delta}}_{1_i}}$ and $\mathbf{C}^{\boldsymbol{\eta}_{1_{i;1}}\boldsymbol{\eta}_{1_{i;1}}}$ is the prediction error variance matrix of $\boldsymbol{\eta}_{1_{i;1}} = \mathbf{Z}_{1_{i;1}}^\top\mathbf{P}_{i\cdot}\mathbf{y}$.

Lastly, the BLUPs of the regression residuals without phenotypic data can be obtained after REML estimation, with:

$$\begin{aligned} \tilde{\boldsymbol{\delta}}_{1_{i;0}} &= \mathbf{G}_{\mathbf{g}_{i;01}}\mathbf{G}_{\mathbf{g}_{i;11}}^{-1}\tilde{\boldsymbol{\delta}}_{1_{i;1}} \\ &= \psi_{1_i}\mathbf{G}_{\mathbf{g}_{i;01}}\mathbf{Z}_{1_{i;1}}^\top\mathbf{P}_{i\cdot}\mathbf{y} \end{aligned} \tag{67}$$

4 Measures of variance explained

This section derives the measures of variance explained for the IFA-LMM. These measures are an extension of Smith et al. (2021) to include known environmental covariates. They consider the variance explained by the regression, that is by the common factors, as an informal measure of model fit. This is because the additive GE effects associated with the common factors, $(\boldsymbol{\Lambda} \otimes \mathbf{I}_v)\mathbf{f}$, are the fitted values in the regression and therefore capture repeatable GEI common to multiple environments. In terms of the IFA-LMM, the additive GE effects can be further partitioned as $(\mathbf{S}\boldsymbol{\Lambda}_\mathbf{s} \otimes \mathbf{I}_v)\mathbf{f}$ and $(\boldsymbol{\Gamma}\boldsymbol{\Lambda}_\mathbf{r} \otimes \mathbf{I}_v)\mathbf{f}$, which capture known and latent sources of repeatable GEI, respectively. The regression residuals, $\boldsymbol{\delta}$, then capture any non-repeatable GEI specific to individual environments.

There are five measures of additive genetic variance explained for the IFA-LMM. The first two measures are used for model selection and the last three are used for interpretation. Note that "additive genetic variance" is shortened to "variance" in the following. Also note that the variance parameters are unknown so they will be replaced by their REML estimates in practice.

1. a. The percentage of variance explained by the known covariates is given by:

$$\begin{aligned}\bar{v}_s &= 100\,\mathrm{cor}\big[(\mathbf{S}\boldsymbol{\Lambda}_\mathbf{s} \otimes \mathbf{I}_v)\mathbf{f}, \mathbf{u}\big]^2 \\ &= 100\,\frac{\mathrm{tr}\big(\mathrm{cov}\big[(\mathbf{S}\boldsymbol{\Lambda}_\mathbf{s} \otimes \mathbf{I}_v)\mathbf{f}, \mathbf{u}\big]\big)^2}{\mathrm{tr}\big(\mathrm{var}\big[(\mathbf{S}\boldsymbol{\Lambda}_\mathbf{s} \otimes \mathbf{I}_v)\mathbf{f}\big]\big)\mathrm{tr}\big(\mathrm{var}\big[\mathbf{u}\big]\big)} \\ &= 100\,\mathrm{tr}\big(\mathrm{var}\big[(\mathbf{S}\boldsymbol{\Lambda}_\mathbf{s} \otimes \mathbf{I}_v)\mathbf{f}\big]\big)/\mathrm{tr}\big(\mathrm{var}[\mathbf{u}]\big) \\ &= 100\,\mathrm{tr}\big(\mathbf{S}\boldsymbol{\Lambda}_\mathbf{s}\mathbf{D}\boldsymbol{\Lambda}_\mathbf{s}^\top\mathbf{S}^\top \otimes \mathbf{G}_\mathbf{g}\big)/\mathrm{tr}\big(\mathbf{G}_\mathbf{e} \otimes \mathbf{G}_\mathbf{g}\big) \\ &= 100\,\mathrm{tr}\big(\mathbf{S}\boldsymbol{\Lambda}_\mathbf{s}\mathbf{D}\boldsymbol{\Lambda}_\mathbf{s}^\top\mathbf{S}^\top\big)/\mathrm{tr}\big(\mathbf{G}_\mathbf{e}\big), \end{aligned} \tag{68}$$

where $\mathbf{f} \sim \mathrm{N}(\mathbf{0}, \mathbf{D} \otimes \mathbf{G}_\mathbf{g})$ and $\mathbf{u} \sim \mathrm{N}(\mathbf{0}, \mathbf{G}_\mathbf{e} \otimes \mathbf{G}_\mathbf{g})$, with $\mathbf{D}$ and $\mathbf{G}_\mathbf{e}$ defined in Equation 25 of the manuscript. Note that $\mathrm{cov}\big[(\mathbf{S}\boldsymbol{\Lambda}_\mathbf{s}\otimes\mathbf{I}_v)\mathbf{f}, \boldsymbol{\Gamma}\boldsymbol{\Lambda}_\mathbf{r}\otimes\mathbf{I}_v)\mathbf{f}\big]\big) = \mathbf{0}$ since the known and latent covariates are orthogonal.

   b. The overall percentage of variance explained by the known and latent covariates is given by:

$$\begin{aligned}\bar{v} &= 100\,\mathrm{cor}\big[(\boldsymbol{\Lambda} \otimes \mathbf{I}_v)\mathbf{f}, \mathbf{u}\big]^2 \\ &= 100\,\mathrm{tr}\big(\mathbf{D}\big)/\mathrm{tr}\big(\mathbf{G}_\mathbf{e}\big), \end{aligned} \tag{69}$$

   where $\boldsymbol{\Lambda} = \mathbf{S}\boldsymbol{\Lambda}_\mathbf{s} + \boldsymbol{\Gamma}\boldsymbol{\Lambda}_\mathbf{r}$.

2. a. The percentage of variance in environment j explained by the known covariates is given by:

$$\begin{aligned}v_{s_j} &= 100\,\mathrm{cor}\big[(\mathbf{S}_j\boldsymbol{\Lambda}_\mathbf{s} \otimes \mathbf{I}_v)\mathbf{f}, \mathbf{u}_j\big]^2 \\ &= 100\,\mathbf{S}_j\boldsymbol{\Lambda}_\mathbf{s}\mathbf{D}\boldsymbol{\Lambda}_\mathbf{s}^\top\mathbf{S}_j^\top/g_{e_j}, \end{aligned} \tag{70}$$

   where $\mathbf{S}_j^\top$ is the q-vector of known covariates, $\mathbf{u}_j = (\boldsymbol{\Lambda}_j\otimes\mathbf{I}_v)\mathbf{f}+\boldsymbol{\delta}_j$ is the vp-vector of additive GE effects and $\boldsymbol{\Lambda}_j^\top$ is the k-vector of factor loadings for the jth environment, with $\mathbf{u}_j \sim \mathrm{N}(0, g_{e_j}\mathbf{G}_\mathbf{g})$ and $g_{e_j} = \boldsymbol{\Lambda}_j\boldsymbol{\Lambda}_j^\top + \psi_j$ which is equal to the jth diagonal of $\mathbf{G}_\mathbf{e}$.

   b. The percentage of variance in environment j explained by the known and latent covariates is given by:

$$\begin{aligned}v_j &= 100\,\mathrm{cor}\big[(\boldsymbol{\Lambda}_j \otimes \mathbf{I}_v)\mathbf{f}, \mathbf{u}_j\big]^2 \\ &= 100\,\boldsymbol{\Lambda}_j\mathbf{D}\boldsymbol{\Lambda}_j^\top/g_{e_j}, \end{aligned} \tag{71}$$

   where $\boldsymbol{\Lambda}_j = \mathbf{S}_j\boldsymbol{\Lambda}_\mathbf{s} + \boldsymbol{\Gamma}_j\boldsymbol{\Lambda}_\mathbf{r}$ and $\boldsymbol{\Gamma}_j^\top$ is the $(p-q)$-vector given by the jth row in $\boldsymbol{\Gamma}$. Note that $\bar{v}_s \neq \sum_{j=1}^p v_{s_j}/p$ and $\bar{v} \neq \sum_{j=1}^p v_j/p$ since the additive GE effects are correlated across environments.

3. a. The percentage of variance explained by known factor l is given by:

$$\begin{aligned}v_{s_l} &= 100\,\mathrm{cor}\big[(\mathbf{S}\boldsymbol{\lambda}_{\mathbf{s}_l} \otimes \mathbf{I}_v)\mathbf{f}, \mathbf{u}\big]^2 \\ &= 100\,d_l\boldsymbol{\lambda}_{\mathbf{s}_l}^\top\mathbf{S}^\top\mathbf{S}\boldsymbol{\lambda}_{\mathbf{s}_l}/\mathrm{tr}\big(\mathbf{G}_\mathbf{e}\big), \end{aligned} \tag{72}$$

   where $\mathbf{f}_l \sim \mathrm{N}(\mathbf{0}, d_l\mathbf{G}_\mathbf{g})$.

   b. The percentage of variance explained by joint factor l is given by:

$$\begin{aligned}v_l &= 100\,\mathrm{cor}\big[(\boldsymbol{\lambda}_l \otimes \mathbf{I}_v)\mathbf{f}, \mathbf{u}\big]^2 \\ &= 100\,d_l/\mathrm{tr}\big(\mathbf{G}_\mathbf{e}\big), \end{aligned} \tag{73}$$

   where $\boldsymbol{\Lambda} = \big[\boldsymbol{\lambda}_1\,\boldsymbol{\lambda}_2\,\ldots\,\boldsymbol{\lambda}_k\big]$ and $\boldsymbol{\lambda}_l = \mathbf{S}\boldsymbol{\lambda}_{\mathbf{s}_l} + \boldsymbol{\Gamma}\boldsymbol{\lambda}_{\mathbf{r}_l}$. Note that $\bar{v}_s = \sum_{l=1}^k v_{s_l}$ and $\bar{v} = \sum_{l=1}^k v_l$ since the additive GE effects are independent across factors.

4. The percentage of variance explained by known covariate i is given by:

$$v_{s_i} = 100\,r_{s_i}^2, \tag{74}$$

   where:
$$\begin{aligned} r_{s_i} &= \mathrm{cor}\big[(\mathbf{s}_i\boldsymbol{\Lambda}_{\mathbf{s}_i} \otimes \mathbf{I}_v)\mathbf{f}, \mathbf{u}\big] \\ &= \frac{\mathbf{s}_i^\top\mathbf{S}\boldsymbol{\Lambda}_\mathbf{s}\mathbf{D}\boldsymbol{\Lambda}_{\mathbf{s}_i}^\top}{\big[\boldsymbol{\Lambda}_{\mathbf{s}_i}\mathbf{D}\boldsymbol{\Lambda}_{\mathbf{s}_i}^\top\big]^{1/2}\,\mathrm{tr}\big(\mathbf{G}_\mathbf{e}\big)^{1/2}}, \end{aligned} \tag{75}$$

   noting that $\mathbf{s}_i^\top\mathbf{s}_i = 1$.
   It then follows that:

$$v_{s_i} = 100\,\frac{\big[\mathbf{s}_i^\top\mathbf{S}\boldsymbol{\Lambda}_\mathbf{s}\mathbf{D}\boldsymbol{\Lambda}_{\mathbf{s}_i}^\top\big]^2}{\big[\boldsymbol{\Lambda}_{\mathbf{s}_i}\mathbf{D}\boldsymbol{\Lambda}_{\mathbf{s}_i}^\top\big]\,\mathrm{tr}\big(\mathbf{G}_\mathbf{e}\big)}. \tag{76}$$

   The measure in Equation 68 can now be written as:

$$\bar{v}_s = 100\,\mathbf{r}_\mathbf{s}^\top\mathbf{R}_\mathbf{ss}^{-1}\mathbf{r}_\mathbf{s}, \tag{77}$$

   where $\mathbf{r}_\mathbf{s} = (r_{s_1}, r_{s_2}, \ldots, r_{s_q})^\top$ and $\mathbf{R}_\mathbf{ss}$ is a $q \times q$ correlation matrix, with elements given by:

$$\begin{aligned} r_{ss_{ij}} &= \mathrm{cor}\big[(\mathbf{s}_i\boldsymbol{\Lambda}_{\mathbf{s}_i} \otimes \mathbf{I}_v)\mathbf{f}, (\mathbf{s}_j\boldsymbol{\Lambda}_{\mathbf{s}_j} \otimes \mathbf{I}_v)\mathbf{f}\big] \\ &= \frac{\mathbf{s}_i^\top\mathbf{s}_j\boldsymbol{\Lambda}_{\mathbf{s}_j}\mathbf{D}\boldsymbol{\Lambda}_{\mathbf{s}_i}^\top}{\big[\boldsymbol{\Lambda}_{\mathbf{s}_i}\mathbf{D}\boldsymbol{\Lambda}_{\mathbf{s}_i}^\top\big]^{1/2}\big[\boldsymbol{\Lambda}_{\mathbf{s}_j}\mathbf{D}\boldsymbol{\Lambda}_{\mathbf{s}_j}^\top\big]^{1/2}}. \end{aligned} \tag{78}$$

   This measure is analogous to the R^2 goodness-of-fit statistic in multiple regression which accounts for multiple (correlated) covariates.

5. a. The percentage of variance in known factor l explained by known covariate i is given by:

$$\begin{aligned} v_{s_{li}} &= 100\,r_{s_{li}}^2 \\ &= 100\,\frac{\big[\mathbf{s}_i^\top\mathbf{S}\boldsymbol{\lambda}_{\mathbf{s}_l}\big]^2}{\boldsymbol{\lambda}_{\mathbf{s}_l}^\top\mathbf{S}^\top\mathbf{S}\boldsymbol{\lambda}_{\mathbf{s}_l}}, \end{aligned} \tag{79}$$

$$\text{where:} \quad r_{s_{li}} = \text{cor}\big[(\mathbf{s}_i\lambda_{s_{li}} \otimes \mathbf{I}_v)\mathbf{f}_l, (\mathbf{S}\boldsymbol{\lambda}_{\mathbf{s}_l} \otimes \mathbf{I}_v)\mathbf{f}_l\big] = \frac{\mathbf{s}_i^\top \mathbf{S}\boldsymbol{\lambda}_{\mathbf{s}_l}}{\big[\boldsymbol{\lambda}_{\mathbf{s}_l}^\top \mathbf{S}^\top \mathbf{S}\boldsymbol{\lambda}_{\mathbf{s}_l}\big]^{1/2}}. \tag{80}$$

The variance explained by all covariates is given by:

$$v_{s_l\cdot} = 100\, \mathbf{r}_{\mathbf{s}_l}^\top \mathbf{R}_{\mathbf{ss}_l}^{-1} \mathbf{r}_{\mathbf{s}_l}, \tag{81}$$

where $\mathbf{r}_{\mathbf{s}_l} = (r_{s_{l1}}, r_{s_{l2}}, \ldots, r_{s_{lq}})^\top$ and $\mathbf{R}_{\mathbf{ss}_l}$ is a $q \times q$ correlation matrix, with elements given by:

$$r_{ss_{lij}} = \mathbf{s}_i^\top \mathbf{s}_j. \tag{82}$$

It then follows that $v_{s_l\cdot} = 100\%$, that is since the known factors are fully explained by the known covariates.

b. The percentage of variance in joint factor l explained by known covariate i is given by:

$$v_{li} = 100\, r_{li}^2 = 100\, [\mathbf{s}_i^\top \boldsymbol{\lambda}_l]^2, \tag{83}$$

$$\text{where:} \quad r_{li} = \text{cor}\big[(\mathbf{s}_i\lambda_{s_{li}} \otimes \mathbf{I}_v)\mathbf{f}_l, (\boldsymbol{\lambda}_l \otimes \mathbf{I}_v)\mathbf{f}_l\big] = \mathbf{s}_i^\top \boldsymbol{\lambda}_l. \tag{84}$$

The variance explained by all covariates is given by:

$$v_{l\cdot} = 100\, \mathbf{r}_l^\top \mathbf{R}_{\mathbf{ss}_l}^{-1} \mathbf{r}_l, \tag{85}$$

where $\mathbf{r}_l = (r_{l1}, r_{l2}, \ldots, r_{lq})^\top$ and $\mathbf{R}_{\mathbf{ss}_l}$ is defined in Equation 82.

It then follows that $v_{l\cdot} = 100\, [\boldsymbol{\lambda}_{\mathbf{s}_l}^\top \mathbf{S}^\top \mathbf{S}\boldsymbol{\lambda}_{\mathbf{s}_l}]$.

References

Gilmour AR, Smith AB, Borg LM, Gogel BJ, Cullis BR (1995) Estimation of factor analytic mixed models for the analysis of multi-treatment multi-environment trial data. Biometrics 51:1440–1450, URL http://doi.org/10.2307/2533274

Heslot N, Akdemir D, Sorrells ME, Jannink JL (2014) Integrating environmental covariates and crop modeling into the genomic selection framework to predict genotype by environment interactions. Theoretical and Applied Genetics 127:463–480, URL http://doi.org/10.1007/s00122-013-2231-5

Jarquín D, Crossa J, Lacaze X, Du Cheyron P, Daucourt J, Lorgeou J, Piraux F, Guerreiro L, Pérez P, Calus M, Burgueño J, de los Campos G (2014) A reaction norm model for genomic selection using high-dimensional genomic and environmental data. Theoretical and Applied Genetics 127:595–607, URL http://doi.org/10.1007/s00122-013-2243-1

Kirkpatrick M, Meyer K (2004) Direct estimation of genetic principal components: Simplified analysis of complex phenotypes. Genetics 168:2295–2306, URL http://doi.org/10.1534/genetics.104.029181

Mazur L (2021) Computational Methods for the Fitting of Factor Analytic Linear Mixed Models with Applications to Plant Variety Trials. PhD thesis, University of Wollongong

Smith A, Norman A, Kuchel H, Cullis B (2021) Plant variety selection using interaction classes derived from factor analytic linear mixed models: Models with independent variety effects. Frontiers in Plant Science 12:737462, URL http://doi.org/10.3389/fpls.2021.737462

Smith AB (1999) Multiplicative mixed models for the analysis of multi-environment trial data. PhD thesis, University of Adelaide, URL http://hdl.handle.net/2440/19539

Smith AB, Borg LM, Gogel BJ, Cullis BR (2019) Estimation of factor analytic mixed models for the analysis of multi-treatment multi-environment trial data. International Biometric Society 24:573–588, URL http://doi.org/10.1007/s13253-019-00362-6

Thompson R, Cullis BR, Smith AB, Gilmour AR (2003) A sparse implementation of the average information algorithm for factor analytic and reduced rank variance models. Australian and New Zealand Journal of Statistics 45:445–459, URL http://doi.org/10.1111/1467-842X.00297

Verbyla AP (1990) A Conditional Derivation of Residual Maximum Likelihood. Australian Journal of Statistics 32:227–230, URL http://dx.doi.org/10.1111/j.1467-842X.1990.tb01015.x
